# Successful bioassay of blood from chronic wasting disease-affected cervids in cervid PrP transgenic *Drosophila*

**DOI:** 10.1186/s40478-026-02372-1

**Published:** 2026-07-15

**Authors:** Alana M. Thackray, Erin E. McNulty, Amy V. Nalls, Marcelo H. Jorge, Michael J. Chamberlain, Mark G. Ruder, Candace K. Mathiason, Raymond Bujdoso

**Affiliations:** 1 Department of Veterinary Medicine, University of Cambridge, Madingley Road, Cambridge, CB3 0ES UK; 2 Department of Microbiology, Immunology and Pathology, Prion Research Center (PRC), Colorado State University, Fort Collins, CO USA; 3 Warnell School of Forestry and Natural Resources, University of Georgia, Athens, GA USA; 4 Southeastern Cooperative Wildlife Disease Study, College of Veterinary Medicine, University of Georgia, Athens, GA USA

**Keywords:** *Drosophila*, Chronic wasting disease, Prion, Infectivity, Blood, Pre-clinical, Transmissible

## Abstract

Chronic wasting disease is a fatal transmissible prion condition of free-ranging and farmed cervids including deer, elk, moose, muntjac and reindeer. Prionemia is a pathogenic feature of chronic wasting disease in cervids and underpins development of an ante-mortem blood test for effective disease management. To help achieve this goal, we show here that cervid PrP transgenic *Drosophila* can detect prion-infected blood from cervids with chronic wasting disease. We first established that cervid PrP *Drosophila* were highly sensitive to cervid prions since these flies were responsive to dilutions in the range of 10^− 2^ to ≥ 10^− 10^ of brain homogenate from terminal chronic wasting disease-affected cervids. We subsequently showed cervid PrP *Drosophila* developed a neurotoxic phenotype, evidenced by accelerated loss of locomotor activity and survival, after exposure to blood from cervids with experimental or natural chronic wasting disease. Cervid PrP *Drosophila* were sensitive to whole blood and blood fractions including plasma, buffy coat and different leukocyte subsets isolated from prion disease-affected cervids. Importantly, we showed that cervid PrP *Drosophila* were sensitive to blood samples isolated from chronic wasting disease-affected cervids during the pre-clinical phase of the condition. Significantly, we demonstrated that the neurotoxic fly phenotype which developed after exposure to prion-infected cervid blood was transmissible but was not associated with the accumulation of RT-QuIC-detectable prion seeding activity, which does occur after exposure to brain-derived cervid prions. Our data show that cervid PrP *Drosophila* can successfully detect blood from CWD-affected cervids and indicate that blood-borne prions have distinct propagation properties in PrP transgenic *Drosophila* compared to brain-derived prions.

**Supplementary Information:**

The online version contains supplementary material available at https://doi.org/10.1186/s40478-026-02372-1.

## Introduction

Chronic wasting disease (CWD) is a fatal transmissible prion condition of cervids including deer, elk, moose, muntjac and reindeer [5, 43, 70]. The condition is prevalent in North America where it is highly contagious in free-ranging and farmed cervids. CWD has also been described in Europe with cases identified in free-ranging cervids located in Finland, Norway and Sweden [4, 65]. In common with other transmissible mammalian prion diseases, such as Creutzfeldt-Jakob disease (CJD) of humans, bovine spongiform encephalopathy (BSE) of cattle, and scrapie of sheep, CWD in cervids is characterised by accumulation of PrPSc, an abnormal conformer of the normal host protein PrPC, in the brain of affected animals [15, 48]. According to the prion hypothesis, the transmissible agent associated with these diseases comprises principally, if not solely, of PrPSc and is referred to as a prion [47]. Since CWD is transmissible from cervids to other animals, at least experimentally [25, 26, 44], concerns have been raised that this prion disease may present a threat to human health through consumption of prion-contaminated cervid products. The zoonotic potential of CWD has been examined in vitro by misfolded protein amplification techniques and in vivo by transmission studies, in non-human primates and human PrP transgenic mice, but remains unresolved [66]. Sensitive diagnostic methods are required to control the spread of CWD in order to alleviate the burden of disease on cervids and to protect human health from possible zoonotic transmission.

The gold standard diagnostic test for cervid CWD is immunohistochemical detection of disease-associated PrP in obex tissue obtained post-mortem, or in peripheral lymphoid tissue isolated either pre- or post-mortem [22, 30, 55, 68]. Although immunohistochemistry has been invaluable as a definitive diagnostic test, alternative more tractable methods of disease detection, other than ELISA and western blot, are required for efficient CWD management [24, 41]. Pathogenesis studies in CWD-affected cervids have shown that PrPSc and prion infectivity accumulate not only in brain and peripheral lymphoid tissue but also within the intestinal tract, muscle and blood, and also in secreta including urine, saliva and faeces [23, 39, 40, 69]. Since blood can be readily collected from live cervids, in a non-invasive and longitudinal manner, it has become a preferred substrate for CWD diagnostic development. Detection of cervid blood-borne prion seeding activity, a surrogate marker of the actual infectious prion, has been achieved by in vitro protein misfolding cyclic amplification (PMCA) [32, 33] and real-time quaking-induced conversion (RT-QuIC) [19, 42]. Collectively, these in vitro amplification studies have shown that prion seeding activity is detectable in whole blood and in association with buffy coat cells from cervids with pre-clinical and clinical experimental or natural CWD, and that the prionemia plateaus relatively early during disease progression. However, detection of hematogenous prions in general is hindered by their low level in blood [9, 34, 45] and in the case of CWD prion seeding activity, appears to be hampered by blood-based inhibitors and differences in the extent of prionemia in cervids with different PrP genotypes [8, 14, 32, 33, 46]. Therefore, while in vitro prion seeding assays have established the potential of a blood-based assay for CWD detection, it is considered that detection of *bona fide* prion infectivity offers a more robust confirmatory approach.

The only reliable method to detect *bona fide* prion infectivity is by bioassay in a suitable experimental host and in the case of hematogenous CWD prions this has been initially addressed by transmission studies in native cervid models [37]. Blood-borne CWD prion infectivity has been detected by transfusion of whole blood from pre-clinical and clinical CWD-infected deer in naïve white-tailed deer [39, 40]. Transmission studies in white-tailed deer also demonstrated that B cells and platelets harbor prion infectivity in the blood of deer with CWD [38]. These observations have been supported by bioassay of blood components from CWD-affected cervids in cervid PrP transgenic mice that showed concordance with transmission studies in the natural host [38]. Although bioassays using mammalian hosts have provided significant advances in understanding the biology of blood-borne cervid prions, these assays are cumbersome, of long duration, expensive and subject to ongoing ethical debate. New tractable prion bioassays are required, in particular those that are reasonably rapid, less expensive and preferably using a less sentient host.

To address this, we have developed *Drosophila* as a new experimental host for the detection of *bona fide* mammalian prion infectivity [12]. We have previously generated *Drosophila* transgenic for white-tailed deer PrP and have shown that adult flies exposed to cervid CWD prions, at the larval stage, showed an increasing severity of a neurotoxic phenotype that was accompanied by a progressive accumulation of prion seeding activity [61]. In addition, head homogenate from cervid PrP *Drosophila* exposed to cervid CWD prions accumulated infectious prions that were transmissible to a mammalian host, namely cervid PrP transgenic mice [61]. Collectively, these observations were indicative of *bona fide* prion replication in cervid PrP *Drosophila*. This view is supported by our previous observations that have demonstrated that PrP transgenic *Drosophila* are permissive for authentic replication of mammalian prions [58].

Our studies presented here show that cervid PrP *Drosophila* were sufficiently sensitive to low levels of brain-derived cervid prions to suggest this novel invertebrate host was capable of the detection of prions in the blood of CWD-affected cervids. This appeared to be the case since we subsequently showed that adult cervid PrP *Drosophila* after exposure, at the larval stage, to blood from cervids with experimental or natural CWD developed a neurotoxic phenotype. Cervid PrP *Drosophila* were sensitive to whole blood and blood fractions including plasma, buffy coat cells and different leukocyte subsets isolated from CWD-affected cervids. Cervid PrP *Drosophila* were capable of the detection of CWD-induced blood-borne toxicity from as few as one hundred buffy coat cells. Significantly, we showed that cervid PrP *Drosophila* could detect blood samples collected from CWD-affected cervids at early time points during pre-clinical disease. The neurotoxic fly phenotype induced by prion-infected cervid blood was transmissible but was not associated with the accumulation of RT-QuIC-detectable prion seeding activity, which does occur after exposure to brain-derived cervid prions. Collectively, our data are consistent with the view that cervid PrP *Drosophila* can be used successfully to bioassay prions in blood from symptomatic and asymptomatic CWD-diseased cervids, and that blood-borne and brain-derived prions have distinct propagation properties in PrP transgenic *Drosophila*.

## Materials and methods

### *Drosophila* fly lines

*Drosophila* transgenic for white-tailed deer PrP (S138 variant, GenBank accession number AF156185, amino acid residues 25–233) were generated by pUASTattB/PhiC31-mediated site-specific transformation [7] as described previously in [61]. The following fly lines were obtained from the Department of Genetics, Cambridge University, UK: *Elav-GAL4* (P{w[+ mW.hs] = GawB}elav[C155]); 51D (w; M{3xP3-RFP.attP}ZH-51D). PrP transgenic *Drosophila* were crossed with the *Elav-GAL4* driver fly line to derive transgenic flies that expressed PrP pan neuronally*.* Non-PrP transgenic 51D *Drosophila* crossed with the *Elav-GAL4* driver fly line were used as control flies where appropriate. All fly lines were raised on standard cornmeal media at 25 ˚C and maintained at low to medium density, and pre-mated before experimental use*.*

### Preparation of *Drosophila* head homogenate

Fly head homogenates were prepared as described previously [58]. Homogenates for Western blot of PrP were prepared by processing 8 female fly heads (cervid PrP *Drosophila* or control 51D *Drosophila*) in 4 µL of lysis buffer [50 mM Tris/HCl, pH 7.5, 100 mM NaCl, 0.5% Nonidet P40 and 1 mM 4-(2-aminoethyl) benzenesulfonyl fluoride] followed by 10 min of sonication on ice.

### SDS-PAGE and western blot

Fly head homogenate was mixed with an equal volume of × 2-strength Laemmli loading buffer, boiled for 10 min, cooled on ice, and then centrifuged at 13,000 g for 5 min at 18 ˚C to remove debris. A volume of sample equivalent to 8 fly heads (cervid PrP *Drosophila* or control 51D *Drosophila*) per track, was subjected to 12% SDS-PAGE run under reducing conditions and Western blot as described in detail previously [60] except that the nitrocellulose membranes were probed with a 1:2000 dilution of anti-PrP monoclonal antibody Sha31 [20]. PrP bands were detected by enhanced chemiluminescence and captured by a Chemidoc MP Imaging System (Bio-Rad).

### Prion inoculation of *Drosophila*

*Drosophila* were exposed at the larval stage of development to whole blood, plasma, buffy coat, peripheral blood mononuclear cells (PBMC) or leukocyte subsets isolated from white-tailed deer with clinical experimental CWD, natural CWD or CWD-negative white-tailed deer, or PBS. In all cases, the same anti-coagulants were used where prion-negative and prion-positive blood samples and blood fractions were compared. Cervid CWD-positive brain homogenate (CBP6) or cervid CWD-negative brain homogenate (cervid #502/504) were used as positive and negative controls, respectively. All cervid inocula were prepared in PBS pH 7.4 and two hundred and fifty microlitres of the relevant samples were added to the top of the cornmeal that contained third instar *Drosophila* larvae in 3-inch plastic vials. Following eclosion (i.e. hatching) flies were transferred to fresh non-treated vials. Supplementary Data 1 shows the list of cervid inocula used in this manuscript. The CWD status for all deer was determined by immunohistochemistry (Colorado State University Veterinary Diagnostic Laboratory) and, on occasion, by prion amplification assay real time quaking induced conversion (RT-QuIC) using peripheral lymphoid tissue as seed. All blood samples from CWD-positive deer were positive when assessed by RT-QuIC whereas those from CWD-negative deer were negative by RT-QuIC.

### Detection of prion seeding activity by RT-QuIC

RT-QuIC was performed as described previously [41]. Substrate consisted of syrian hamster recombinant PrP 0.1 mg/mL in RT-QuIC buffer (320 mM NaCL, 1.0 mM EDTA, 10 µM Thioflavin T (ThT) [27]. Seed was *Drosophila* head homogenate prepared as described above and diluted 1:10 in 0.1% SDS. Reaction mixes comprised 98 µL of substrate and 2 µL of seed prepared in quadruplicate on two plates by two investigators (8 replicates/sample). Reaction mixes that contained known RT-QuIC positive or negative PrP *Drosophila* head homogenates as seed were included as positive and negative controls, respectively, on all plates (10 replicates/sample). Reaction mix plates were placed in a BMG Labtech Polarstar™ fluorometer and subjected to 700 rpm double-orbital shaking for 1 min every other minute for 15 min for 250 cycles at 55 ˚C. After each cycle ThT fluorescence was read at an excitation of 450 nm and emission of 480 nm with the gain at 1700 and using orbital averaging with 20 flashes per well with a 4 mm setting. Fluorescent readings were recorded for all sample reactions for a total time of 62 h at a temperature of 55 °C. Samples were considered positive if they crossed a threshold (5 SD above the mean of the initial 5 readings). The inverse of the time when the reaction reached the threshold (1/time to threshold) was used to determine amyloid formation rate [54].

### Negative geotaxis climbing assay

The locomotor ability of flies was assessed in a negative geotaxis climbing assay [67], initiated with 45 (3 × n = 15) age-matched, pre-mated female flies from one generation in each treatment group [63]. Locomotor ability, expressed as mean performance index (PI) (± SD) at individual time points for each treatment group was plotted as a regression line.

### Survival assay

Survival of *Drosophila* was assessed and recorded as previously described [59]. Survival curves are shown as Kaplan–Meier plots.

### Statistical analysis

Statistical analyses of the RT-QuIC data were performed using either a Mann–Whitney test when there were non-zero values in the negative controls, or a Wilcoxon signed-rank test when there were only zero values in the negative controls. Statistical analysis of the negative geotaxis climbing assay data was performed using an unpaired (two-tailed) Student t test. The Log-rank (Mantel-Cox) test was used to assess the survival assay data. Statistical analysis of median survival times was carried out using Kaplan–Meier plots. All statistical analyses were performed using Prism Version 10.3.0 or 10.6.0 (GraphPad Software Inc, San Diego, USA) where each CWD-positive treatment group was compared to its respective CWD-negative treatment group.

## Results

### Sensitive detection of cervid brain-derived CWD prions by cervid PrP *Drosophila*

**Fig. 1:**
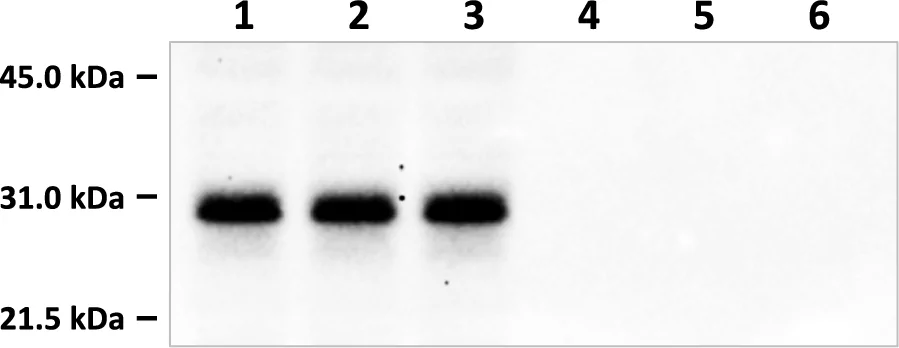
Western blot detection of prion protein expression in cervid PrP *Drosophila*. *Elav* x cervid PrP or *Elav* x 51D *Drosophila* were harvested at 5 days of age. Fly head homogenates were prepared and analysed by SDS-PAGE and Western blot with anti-PrP monoclonal antibody Sha31 as described in the Materials and Methods. Molecular mass markers (kDa) are shown on the left-hand side. Tracks 1–3: *Elav* x cervid PrP *Drosophila*; Tracks 4–6: *Elav* x 51D *Drosophila*

Here we have determined the potential of *Drosophila* transgenic for cervid (white-tailed deer) PrP, that we have generated previously [61], as a novel in vivo bioassay host for use in the detection of blood from CWD-infected cervids. Invertebrates including *Drosophila* do not normally express PrP or possess an orthologue of the mammalian PrP gene in their genome. The Western blot in Fig. 1 shows that cervid PrP is efficiently expressed in *Drosophila* in a manner like other mammalian forms of PrP expressed in the fly [64].

**Fig. 2:**
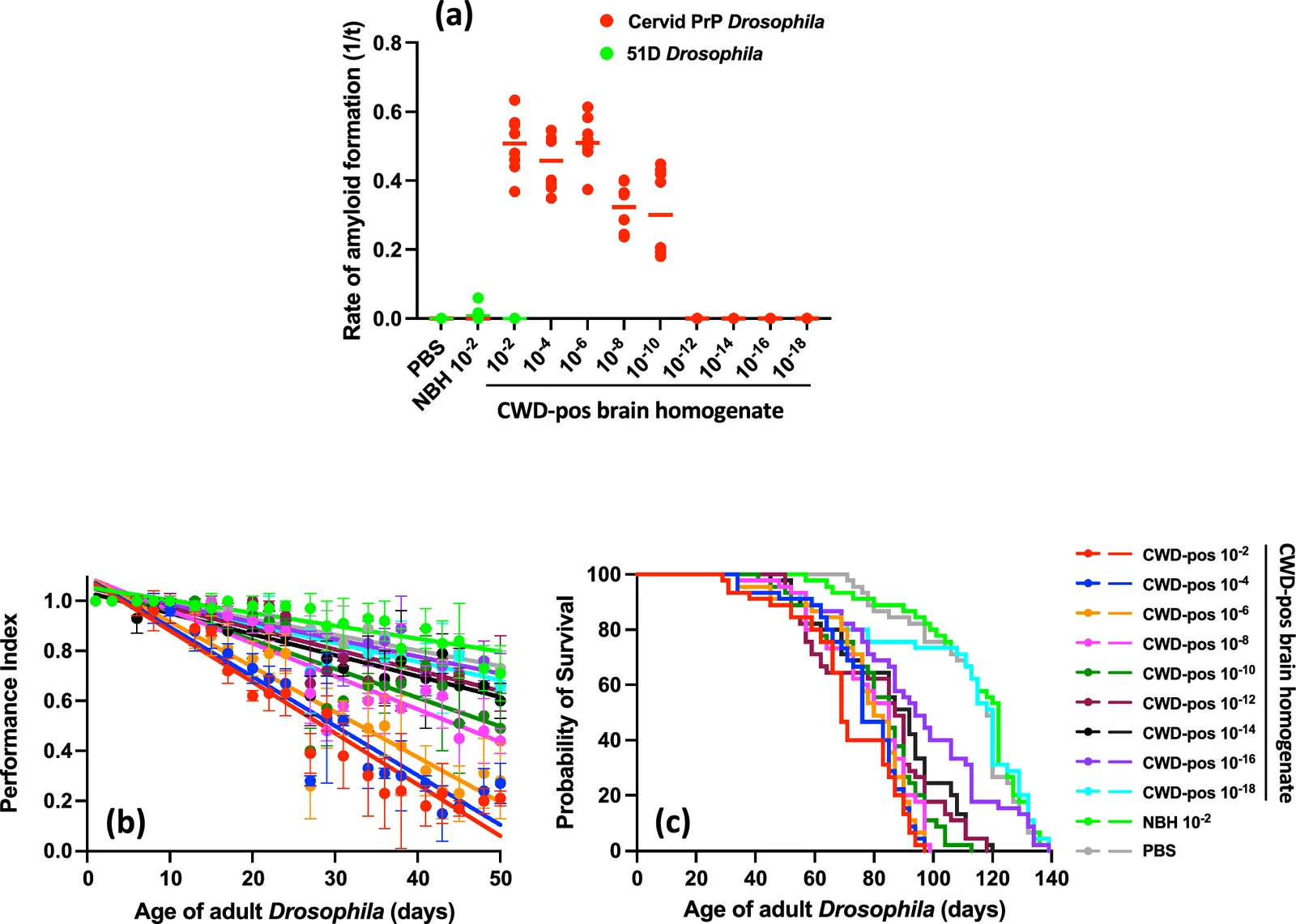
Sensitivity of cervid PrP *Drosophila* to brain-derived CWD prions. *Elav* x cervid PrP or *Elav* x 51D *Drosophila* were exposed to a 10^–2^ dilution series of experimentally infected CWD-positive white-tailed deer brain homogenate (CBP6), or a 10^–2^ dilution of CWD-negative white-tailed deer normal brain homogenate (#502/504), or PBS at the larval stage. After hatching, adult *Drosophila* were assessed for the response to cervid CWD prion exposure. **a** *Accumulation of prion seeding activity*. Adult *Drosophila* were collected at 40-days of age when head homogenate was prepared for use as seed in RT-QuIC reactions. The data shown are rate of amyloid formation (1/t) for each treatment group. **b** *Assessment of locomotor activity*. *Elav* x cervid PrP adult *Drosophila* were assessed for locomotor activity by a negative geotaxis climbing assay. The mean performance index (± SD), expressed as a regression line, is shown for three groups of n = 15 flies per time point. **c** *Assessment of survival*. After hatching, the number of surviving adult *Elav* x cervid PrP flies was recorded three times a week and the data shown as Kaplan-Meier plots. Statistical analysis of Fig. 2 is shown in Supplementary Data 2. *Abbreviations:* CWD-pos: experimentally infected CWD-positive white-tailed deer brain homogenate (CBP6); NBH: Prion-free white-tailed deer normal brain homogenate; PBS: phosphate buffered saline

We first investigated the sensitivity of cervid PrP *Drosophila* to CWD prions as shown by the data in Fig. 2. *Drosophila* were exposed, at the larval stage, to a 10^–2^ dilution series of CWD-infected cervid brain homogenate or a 10^–2^ dilution of prion-free cervid normal brain homogenate (NBH). Subsequently, after hatching, adult *Drosophila* were assessed in vitro for accumulation of prion seeding activity, a surrogate marker of *bona fide* prion infectivity [3], and in vivo for development of a neurotoxic phenotype [59, 63]. Adult cervid PrP *Drosophila* accumulated prion seeding activity after exposure to dilutions of 10^–2^—10^–10^ of inoculum with no response seen following exposure to dilutions of 10^–12^—10^–18^ of inoculum (Fig. 2a). Cervid PrP *Drosophila* displayed a neurotoxic phenotype evidenced by significant accelerated decline in locomotor ability (Fig. 2b) and significant accelerated loss of survival (Fig. 2c) after exposure to dilutions of 10^–2^—≥ 10^–14^ of inoculum. Statistical analysis of Fig. 2 is shown in Supplementary Data 2.

These data show that cervid PrP *Drosophila* demonstrate a high sensitivity to brain-derived cervid prions. Furthermore, the concomitant occurrence of prion seeding activity and neurotoxic phenotype in cervid prion exposed cervid PrP *Drosophila* is indicative of *bona fide* prion replication in these flies, which has been shown to occur [61]. 

### Detection of blood samples from CWD-affected cervids by cervid PrP *Drosophila*

We next investigated the ability of cervid PrP *Drosophila* to identify blood samples collected from cervids with terminal CWD induced either experimentally or by natural exposure to the condition. Accordingly, *Drosophila* were exposed at the larval stage to either whole blood or various blood fractions from CWD-affected or prion-free control cervids. After hatching, adult *Drosophila* were assessed for locomotor ability and survival.

**Fig. 3:**
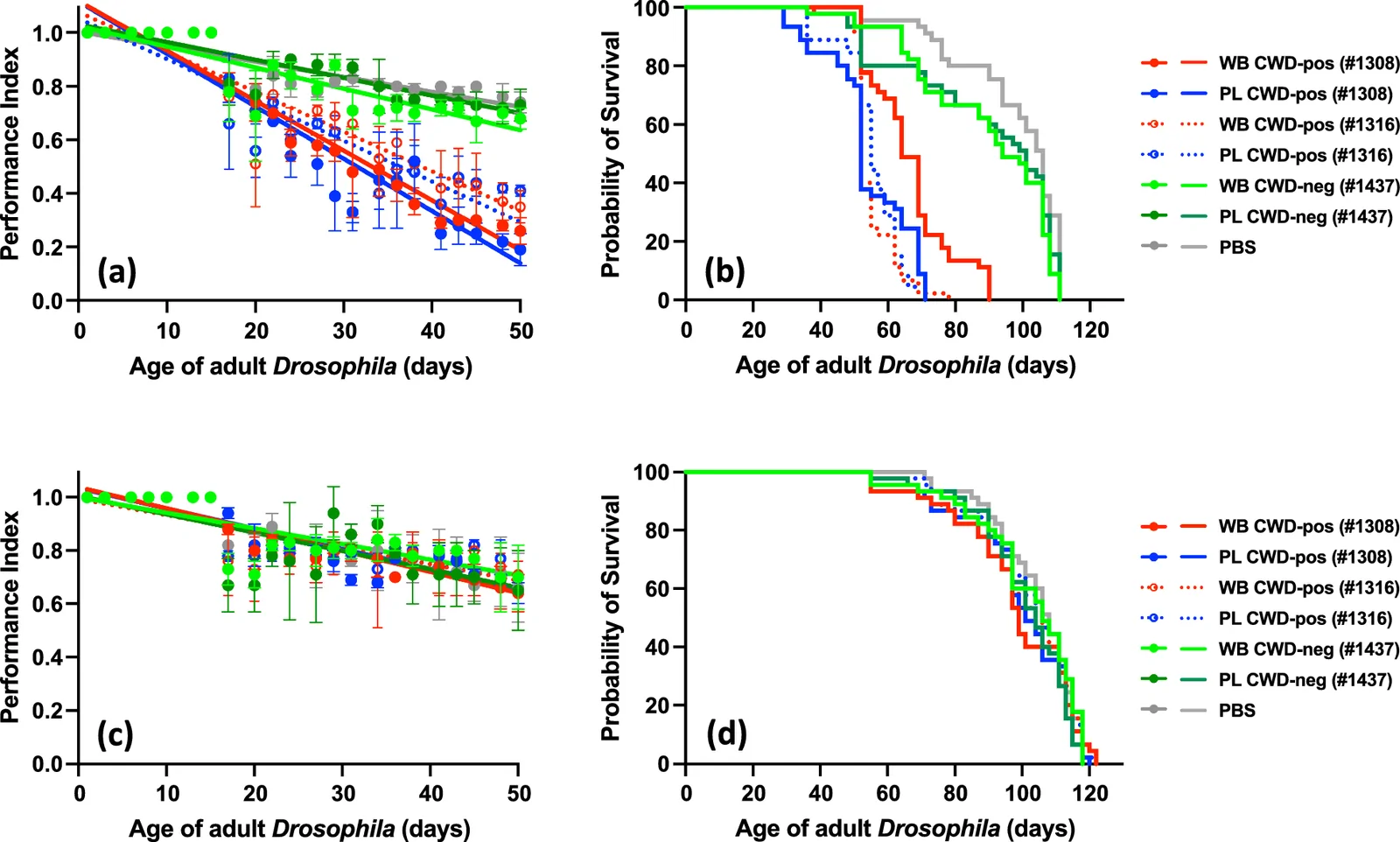
Cervid PrP *Drosophila* exposed to whole blood and plasma from cervids with experimental CWD. *Elav* x cervid PrP *Drosophila* (**a**) and (**b**) and *Elav* x 51D *Drosophila* (**c**) and (**d**) were exposed to a 10^–2^ dilution of whole blood or plasma from clinical experimentally infected CWD-positive or prion-free white-tailed deer, or PBS, at the larval stage. After hatching, adult *Drosophila* were assessed for locomotor activity by a negative geotaxis climbing assay (**a**) and (**c**) or for survival (**b**) and (**d**). The mean performance index (± SD), expressed as a regression line, is shown for three groups of n = 15 flies of each genotype per time point (**a**) and (**c**). For the survival assay the number of surviving flies was recorded three times a week and the data shown as Kaplan–Meier plots (**b**) and (**d**). Individual animal numbers are shown in brackets on each key. # = cervid identification number. Statistical analysis of Fig. 3 is shown in Supplementary Data 3. *Abbreviations:* WB: whole blood; PL: plasma; CWD-pos: CWD positive; CWD-neg: CWD negative; PBS: phosphate buffered saline

Figure 3 shows that exposure of cervid PrP *Drosophila* to whole blood or plasma from experimental CWD-infected cervids resulted in a significant accelerated decline in locomotor activity (Fig. 3a) and survival (Fig. 3b) compared to the response seen with control inoculum. In contrast, control 51D *Drosophila* showed no difference in locomotor activity (Fig. 3c) or survival (Fig. 3d) following exposure to the same blood samples. Statistical analysis of Fig. 3 is shown in Supplementary Data 3.

**Fig. 4:**
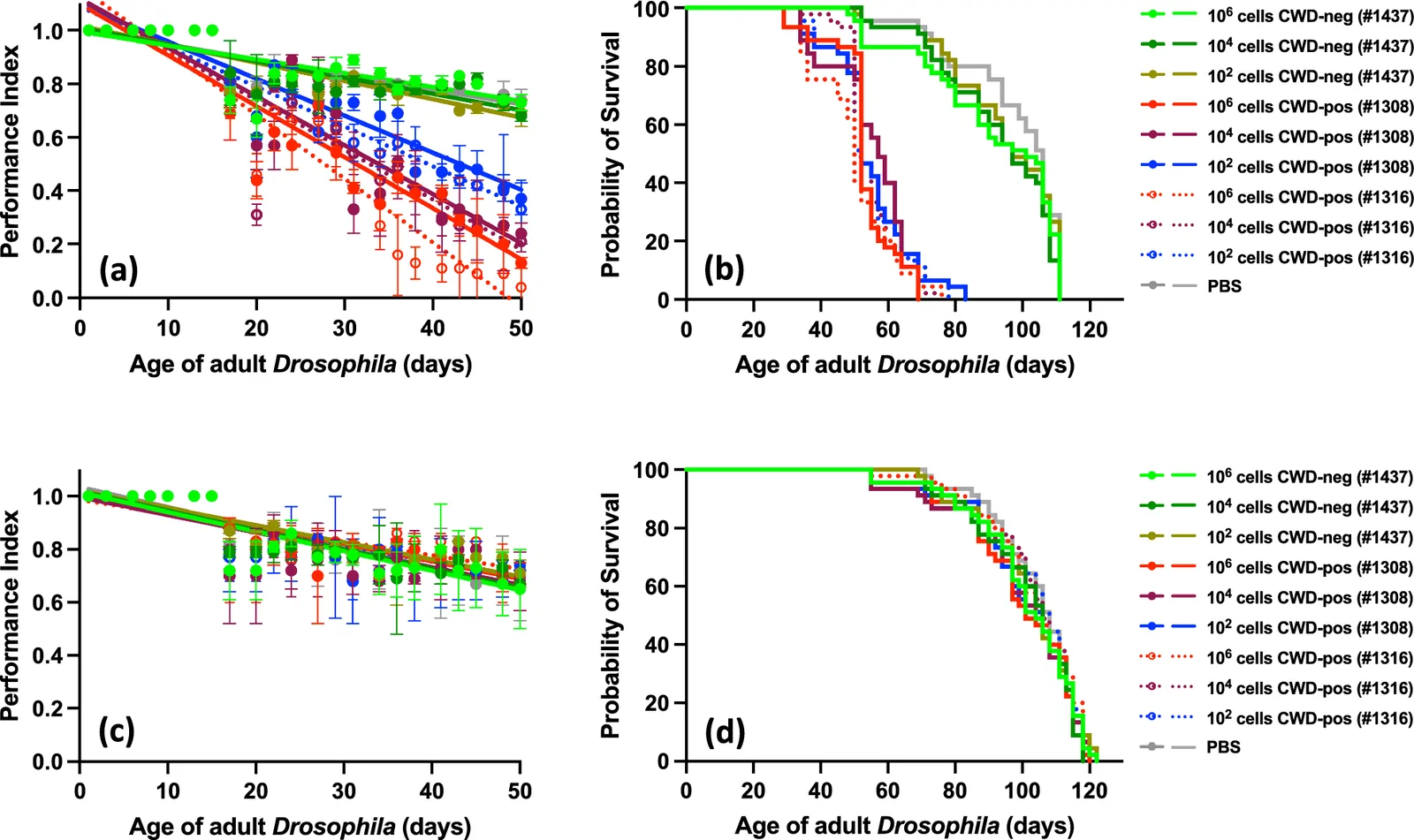
Cervid PrP *Drosophila* exposed to buffy coat cells from cervids with experimental CWD. *Elav* x cervid PrP *Drosophila* (**a**) and (**b**) and *Elav* x 51D *Drosophila* (**c**) and (**d**) were exposed to 10^2^, 10^4^ or 10^6^ buffy coat cells from clinical experimentally infected CWD-positive, or prion-free white-tailed deer, or PBS, at the larval stage. After hatching, adult *Drosophila* were assessed for locomotor activity by a negative geotaxis climbing assay (**a**) and (**c**) or for survival (**b**) and (**d**). The negative geotaxis mean performance index (± SD), expressed as a regression line, is shown for three groups of n = 15 flies of each genotype per time point (**a**) and (**c**). For the survival assay the number of surviving flies was recorded three times a week and the data shown as Kaplan–Meier plots (**b**) and (**d**). Individual animal numbers are shown in brackets on each key. # = cervid identification number. Statistical analysis of Fig. 4 is shown in Supplementary Data 4. *Abbreviations:* CWD-neg: CWD negative; CWD-pos: CWD positive; PBS: phosphate buffered saline

The data in Fig. 4 demonstrate that cervid PrP *Drosophila* showed an accelerated decline in locomotor activity following exposure to buffy coat cells from experimental CWD-infected cervids compared to control buffy coat cells (Fig. 4a). This response diminished with decreasing number of buffy coat cells in the inoculum and was statistically significant with ≥ 100 cells for one of the two animals tested. Cervid PrP *Drosophila* also showed an accelerated loss in survival following exposure to buffy coat cells from experimental CWD-infected cervids compared to control material (Fig. 4b). In contrast, control 51D *Drosophila* showed no difference in locomotor activity (Fig. 4c) or survival (Fig. 4d) following exposure to the same buffy coat cell samples. Statistical analysis of Fig. 4 is shown in Supplementary Data 4.

**Fig. 5:**
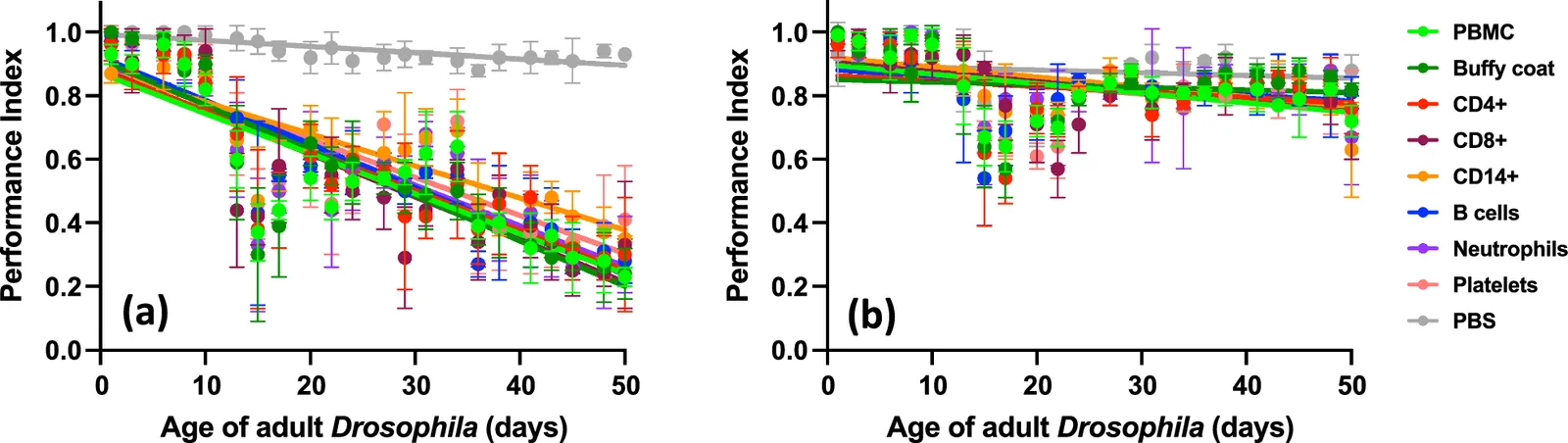
Cervid PrP *Drosophila* exposed to leukocyte subsets from cervids with experimental CWD. Adult *Elav* x cervid PrP *Drosophila* were assessed for locomotor activity by a negative geotaxis climbing assay after exposure at the larval stage to homogenate prepared from 10^**5**^ cells of various blood leukocyte populations prepared from **a** clinical experimentally infected CWD-positive white-tailed deer (cervid #1528 for all samples except CD8^**+**^ cells which were from cervid #1697) or **b** prion-free white-tailed deer (cervid #1531), or PBS (**a**) and (**b**). The negative geotaxis mean performance index (± SD), expressed as a regression line, is shown for three groups of n = 15 flies of each genotype per time point. Statistical analysis of Fig. 5 is shown in Supplementary Data 5. *Abbreviations:* PBMC: peripheral blood mononuclear cells; PBS: phosphate buffered saline

We investigated the range of buffy coat cell subsets in the blood of CWD-affected cervids that induced toxicity in cervid PrP *Drosophila*. The data in Fig. 5 show that FACS purified B cells, T cells (CD4^**+**^ / CD8^**+**^), macrophages (CD14^**+**^), neutrophils and platelets from the blood of CWD-affected cervids induced an accelerated loss of locomotor ability (Fig. 5a) compared to the response seen with prion-free control cells (Fig. 5b). Statistical analysis of Fig. 5 is shown in Supplementary Data 5.

Supplementary Data 6 shows that exposure of cervid PrP *Drosophila* to plasma (either platelet-rich or cell-free) from experimental CWD-infected cervids resulted in a significant accelerated decline in locomotor activity (Supplementary Data 6a) compared to the response seen with control inoculum (Supplementary Data 6b). Statistical analysis of Supplementary Data 6 is shown in Supplementary Data 7.

**Fig. 6:**
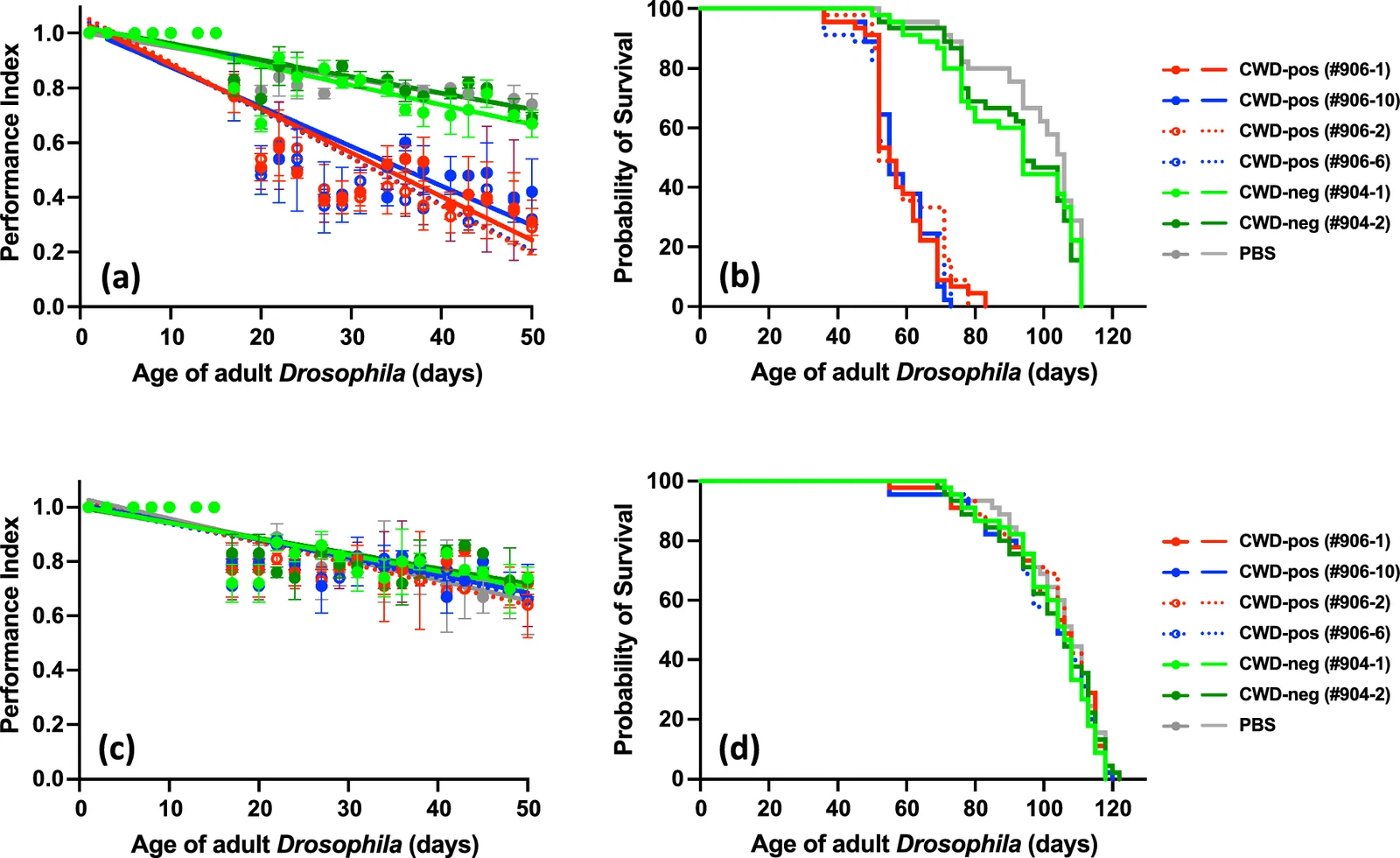
Cervid PrP *Drosophila* exposed to whole blood from cervids with natural CWD. *Elav* x cervid PrP *Drosophila* (**a**) and (**b**) and *Elav* x 51D *Drosophila* (**c**) and (**d**) were exposed to a 10^**–2**^ dilution of whole blood from cervids with natural exposure to CWD, or from prion-free white-tailed deer, or PBS, at the larval stage. After hatching, adult *Drosophila* were assessed for locomotor activity by a negative geotaxis climbing assay (**a**) and (**c**) or for survival (**b**) and (**d**). The negative geotaxis mean performance index (± SD), expressed as a regression line, is shown for three groups of n = 15 flies of each genotype per time point (**a**) and (**c**). For the survival assay the number of surviving flies was recorded three times a week and the data shown as Kaplan–Meier plots (**b**) and (**d**). Individual animal numbers are shown in brackets on each key. # = cervid identification number. Statistical analysis of Fig. 6 is shown in Supplementary Data 8. *Abbreviations:* CWD-pos: CWD positive; CWD-neg: CWD negative; PBS: phosphate buffered saline

The data in Fig. 6 show that exposure of cervid PrP *Drosophila* to whole blood from healthy-appearing cervids with natural CWD resulted in a significant accelerated decline in locomotor activity (Fig. 6a) and significant accelerated loss of survival (Fig. 6b) compared to that seen in response to control inoculum. In contrast, control 51D *Drosophila* showed no difference in locomotor activity (Fig. 6c) or survival (Fig. 6d) following exposure to the same blood samples. Statistical analysis of Fig. 6 is shown in Supplementary Data 8.

### Detection of pre-clinical CWD-infected cervid blood by cervid PrP *Drosophila*

Our data so far have shown that cervid PrP *Drosophila* can detect blood samples from cervids with clinical CWD. We next determined whether this invertebrate host could also detect blood samples from cervids with pre-clinical CWD. To do so, cervid PrP *Drosophila*, at the larval stage, were exposed to blood samples isolated from white-tailed deer prior to the development of clinical signs of CWD induced experimentally or by natural exposure.

**Fig. 7:**
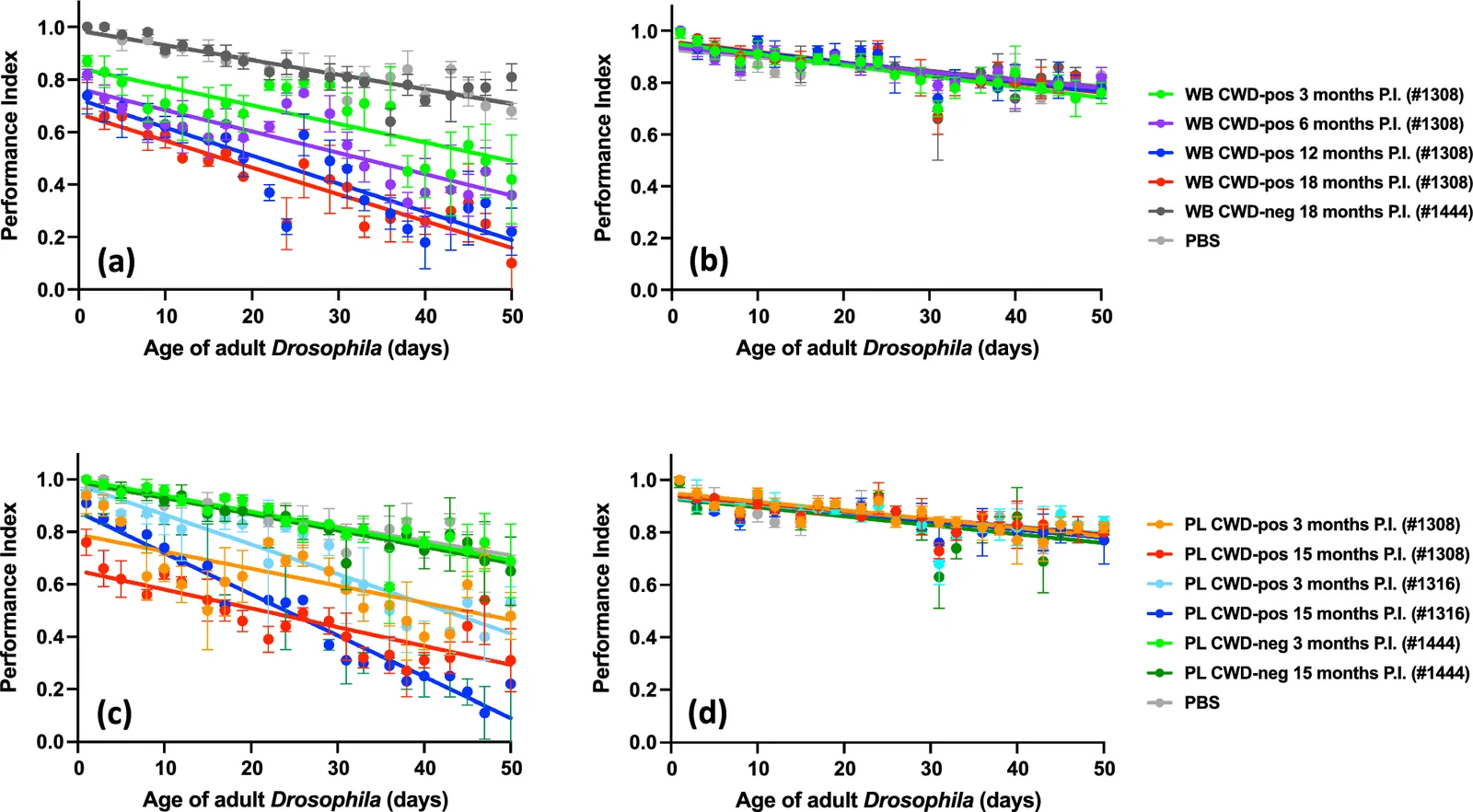
Cervid PrP *Drosophila* exposed to whole blood and plasma from cervids with pre-clinical experimental CWD. *Elav* x cervid PrP *Drosophila* (**a**) and (**c**) and *Elav* x 51D *Drosophila* (**b**) and (**d**) were exposed to a 10^**–2**^ dilution of whole blood (**a**) and (**b**) or plasma (**c**) and (**d**) from experimentally infected CWD-positive, or from prion-free white-tailed deer, or PBS, at the larval stage. After hatching, adult *Drosophila* were assessed for locomotor activity by a negative geotaxis climbing assay. The negative geotaxis mean performance index (± SD), expressed as a regression line, is shown for three groups of n = 15 flies of each genotype per time point. Experimentally infected CWD-positive white-tailed deer animal #1308 was used for whole blood analysis in (**a**) and (**b**). Experimentally infected CWD-positive white-tailed deer animals #1308 (orange and red circles) and #1316 (light blue and dark blue circles) used for plasma analysis in (**c**) and (**d**). Deer #1308 and #1316 developed clinical signs of disease at 18 months P.I. and 15 months P.I. respectively. Whole blood and plasma from prion-free white-tailed deer animal #1444 used in (**a**) and (**b**), and (**c**) and (**d**), respectively. Figure 7d has the 15-month post infection CWD-negative plasma sample and not the 3-month equivalent. Animal numbers are shown in brackets on each key. # = cervid identification number. Statistical analysis of Fig. 7 is shown in Supplementary Data 9. *Abbreviations:* WB: whole blood; PL: plasma; CWD-pos: CWD-positive; CWD-neg: CWD-negative; PBS: phosphate buffered saline; P.I.: post inoculation

Figure 7 shows that cervid PrP *Drosophila* displayed a significant accelerated decline in locomotor activity after exposure to time-course whole blood (Fig. 7a) or plasma (Fig. 7c) from cervids with experimental CWD compared to the response seen with control samples. The decline in locomotor activity was more pronounced when cervid PrP *Drosophila* were exposed to either whole blood or plasma isolated during the later stages of the time course of the experimental CWD infection. The earliest time point that cervid PrP *Drosophila* were able to detect known prion infected blood samples from experimental CWD-affected cervids was ≥ 3 months post CWD-infection. In contrast, control 51D *Drosophila* showed no difference in decline in locomotor activity following exposure to the same time course whole blood samples (Fig. 7b) or plasma (Fig. 7d). These data show that cervid PrP *Drosophila* can detect blood samples from asymptomatic experimental CWD-infected cervids since clinical signs of disease in these animals are typically not evident until approximately 15–24 months post infection [43]. Statistical analysis of Fig. 7 is shown in Supplementary Data 9.

**Fig. 8:**
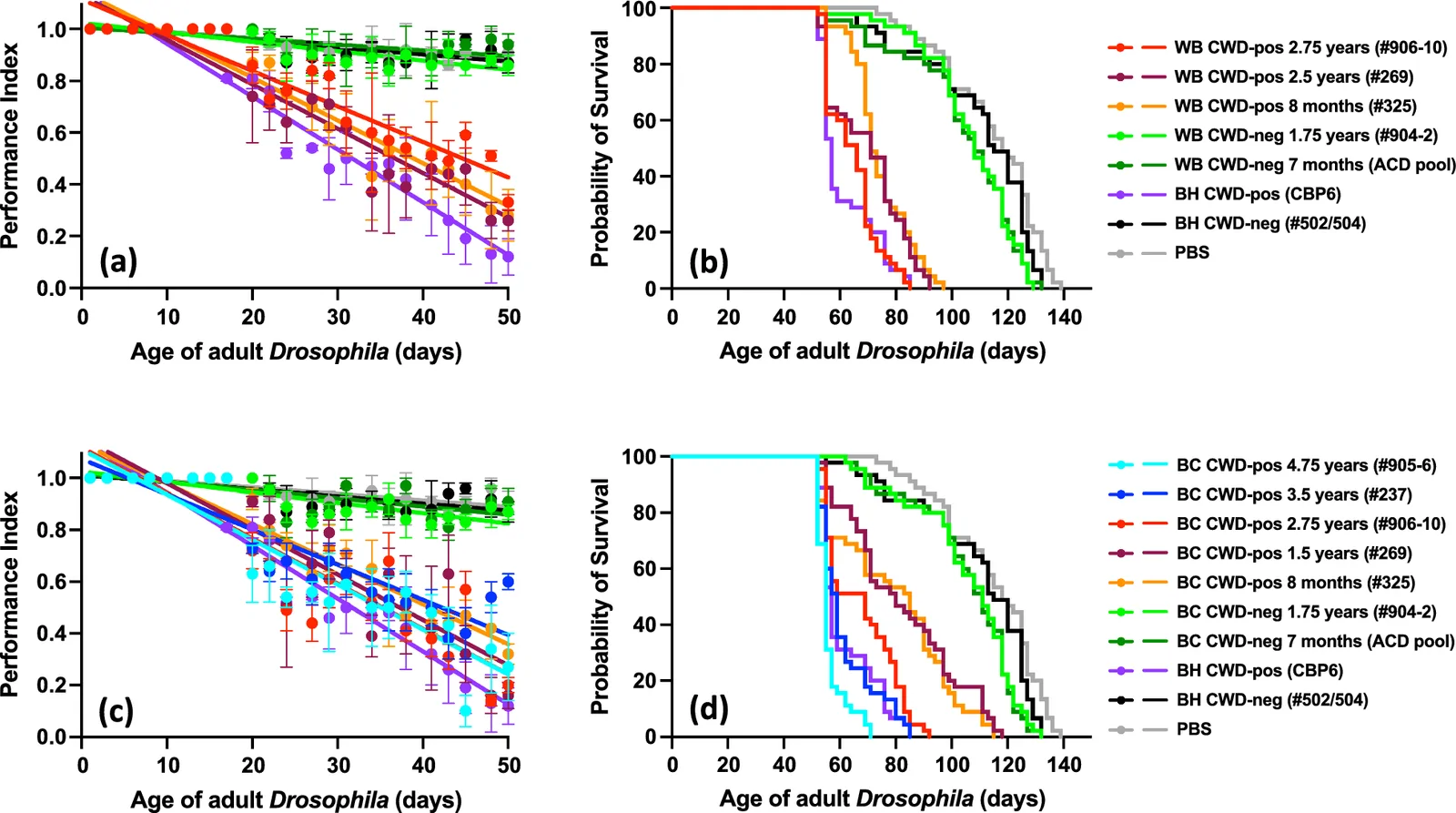
Cervid PrP *Drosophila* exposed to time course blood fractions from cervids with natural CWD. *Elav* x cervid PrP *Drosophila* were exposed to a 10^–2^ dilution of whole blood (**a**) and (**b**) or 10^6^ buffy coat cells (**c**) and (**d**) from white-tailed deer with natural-exposure to CWD, or from prion-free white-tailed deer, or PBS, at the larval stage. After hatching, adult *Drosophila* were assessed for locomotor activity by a negative geotaxis climbing assay (**a**) and (**c**) or for survival (**b**) and (**d**). The negative geotaxis mean performance index (± SD), expressed as a regression line, is shown for three groups of n = 15 flies per time point (**a**) and (**c**). For the survival assay the number of surviving flies was recorded three times a week and the data shown as Kaplan–Meier plots (**b**) and (**d**). Animal numbers are shown in brackets on each key. # = cervid identification number. Statistical analysis of Fig. 8 is shown in Supplementary Data 10. *Abbreviations:* WB: whole blood; BH: brain homogenate; BC: buffy coat; CWD-pos: CWD-positive; CWD-neg: CWD-negative; PBS: phosphate buffered saline; ACD pool: pooled samples from a number of prion-free white-tailed deer (whole blood or buffy coat as shown)

We next investigated the ability of cervid PrP transgenic *Drosophila* to detect blood samples harvested at defined-age time-points from cervids with natural CWD infection. The data in Fig. 8 show that cervid PrP *Drosophila* displayed a significant accelerated decline in locomotor activity (Fig. 8a) and significant accelerated loss of survival (Fig. 8b) after exposure to whole blood samples from cervids with natural CWD at 8–33 months of age compared to similar aged-matched control samples. The data in Fig. 8 also demonstrate that cervid PrP transgenic *Drosophila* showed a statistically significant accelerated decline in locomotor activity (Fig. 8c) and significant accelerated loss of survival (Fig. 8d) after exposure to buffy coat cells isolated from cervids with natural CWD at 8–57 months of age compared to similar aged control samples (Fig. 8d). Statistical analysis of Fig. 8 is shown in Supplementary Data 10. In contrast, control 51D *Drosophila* showed no difference in decline in locomotor activity or loss of survival following exposure to the same blood samples (Supplementary Data 11). The data in Fig. 8 show that cervid PrP *Drosophila* can detect blood samples from cervids with asymptomatic natural CWD infection since clinical signs of disease in these animals are typically not evident until approximately 24 months post infection [43]. Statistical analysis of Supplementary Data 11 is shown in Supplementary Data 12.

### Lack of detectable RT-QuIC prion seeding activity in CWD blood-exposed cervid PrP *Drosophila*

We have shown that cervid PrP *Drosophila* develop a neurotoxic phenotype after exposure to blood samples from CWD-affected cervids. We used RT-QuIC to assess whether this response was associated with the accumulation of prion seeding activity. Accordingly, head homogenate was prepared from adult cervid PrP *Drosophila* exposed, at the larval stage, to whole blood or blood fractions from cervids with experimental or natural CWD-infection as described above in Figs. 3, 4, 5, 6, 7 and 8. No prion seeding activity was observed in any head homogenate from cervid PrP *Drosophila* exposed to any of the cervid blood samples analysed (data not shown).

### Transmissible PrP-dependent toxic moiety is present in CWD-affected cervid blood

We investigated whether the neurotoxic fly phenotype induced by blood samples from CWD-affected cervids was transmissible through fly-to-fly transmission. To do so, we prepared head homogenate from 40-day old cervid PrP *Drosophila*, or control 51D *Drosophila*, that had been exposed at the larval stage to whole blood or brain material from donor cervids with clinical CWD (primary passage *Drosophila*). These head homogenates were subsequently used to inoculate fresh batches of recipient cervid PrP *Drosophila* larvae (secondary passage *Drosophila*). After hatching, the locomotor ability of secondary passage cervid PrP *Drosophila* was assessed by a negative geotaxis climbing assay. In addition, we prepared head homogenate from 5- and 40-day old secondary passage cervid PrP *Drosophila* that were used as seed in RT-QuIC reactions to assess prion seeding activity.

**Fig. 9:**
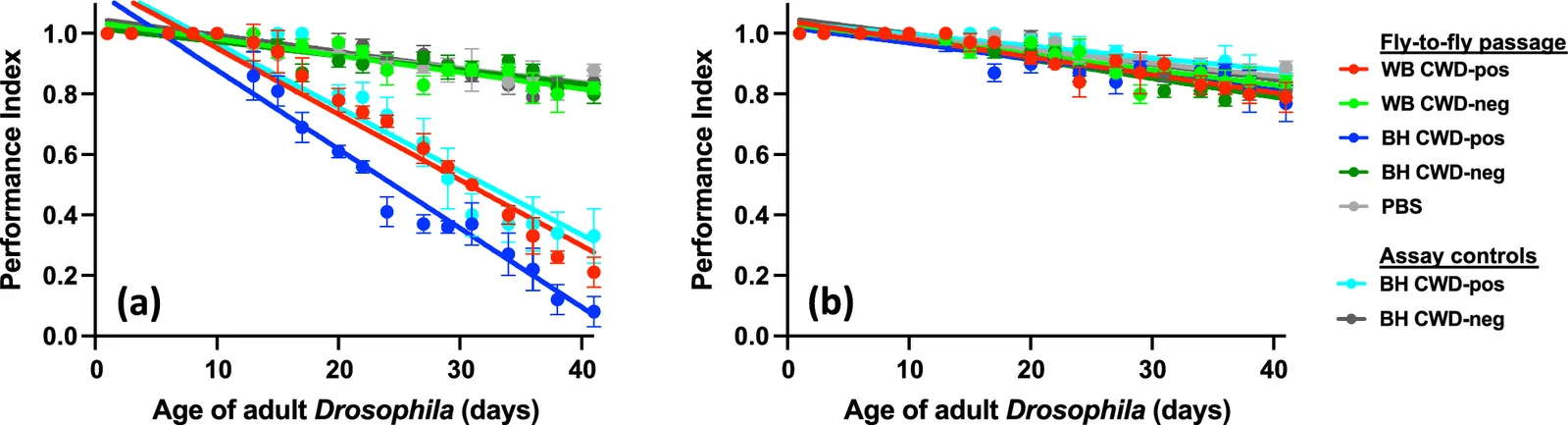
Transmissible toxic moiety is present in CWD-infected cervid blood. Cervid whole blood and brain homogenate was passaged in *Elav* x cervid PrP *Drosophila* or *Elav* x 51D *Drosophila* (primary passage). The inocula at primary passage were the following: whole blood from a white-tailed deer with clinical experimental CWD; whole blood from a prion-free white-tailed deer; cervid CWD-positive brain homogenate (CBP6); brain homogenate from a prion-free cervid; or PBS. Head homogenate was prepared from 40-day old primary passage *Drosophila* and subsequently used to inoculate a fresh batch of *Elav* x cervid PrP *Drosophila* (secondary passage). After hatching, a negative geotaxis climbing assay was performed on secondary passage adult flies derived from primary passage in *Elav* x cervid PrP *Drosophila* (a) or from *Elav* x 51D *Drosophila* (b). The negative geotaxis mean performance index (± SD), expressed as a regression line, is shown for three groups of n = 15 flies per time point. Statistical analysis of Fig. 9 is shown in Supplementary Data 13. *Abbreviations:* WB CWD-pos: cervid CWD-positive whole blood; WB CWD-neg: prion-free white-tailed deer whole blood; BH CWD-pos: cervid CWD-positive brain homogenate (CBP6); BH CWD-neg: prion-free white-tailed deer brain homogenate; PBS: phosphate buffered saline

The data in Fig. 9 show the locomotor ability of secondary passage cervid PrP *Drosophila* after exposure to head homogenate from primary passage *Drosophila*. Head homogenate from CWD-whole blood-exposed and CWD-infected cervid brain material-exposed primary passage cervid PrP *Drosophila* induced a significant accelerated decline in locomotor ability in recipient flies compared to that seen with control primary passage cervid PrP *Drosophila* head homogenate (Fig. 9a). In contrast, no difference was seen in secondary passage cervid PrP *Drosophila* that were exposed to head homogenate from primary passage control 51D *Drosophila* (Fig. 9b). Statistical analysis of Fig. 9 is shown in Supplementary Data 13.

**Fig. 10:**
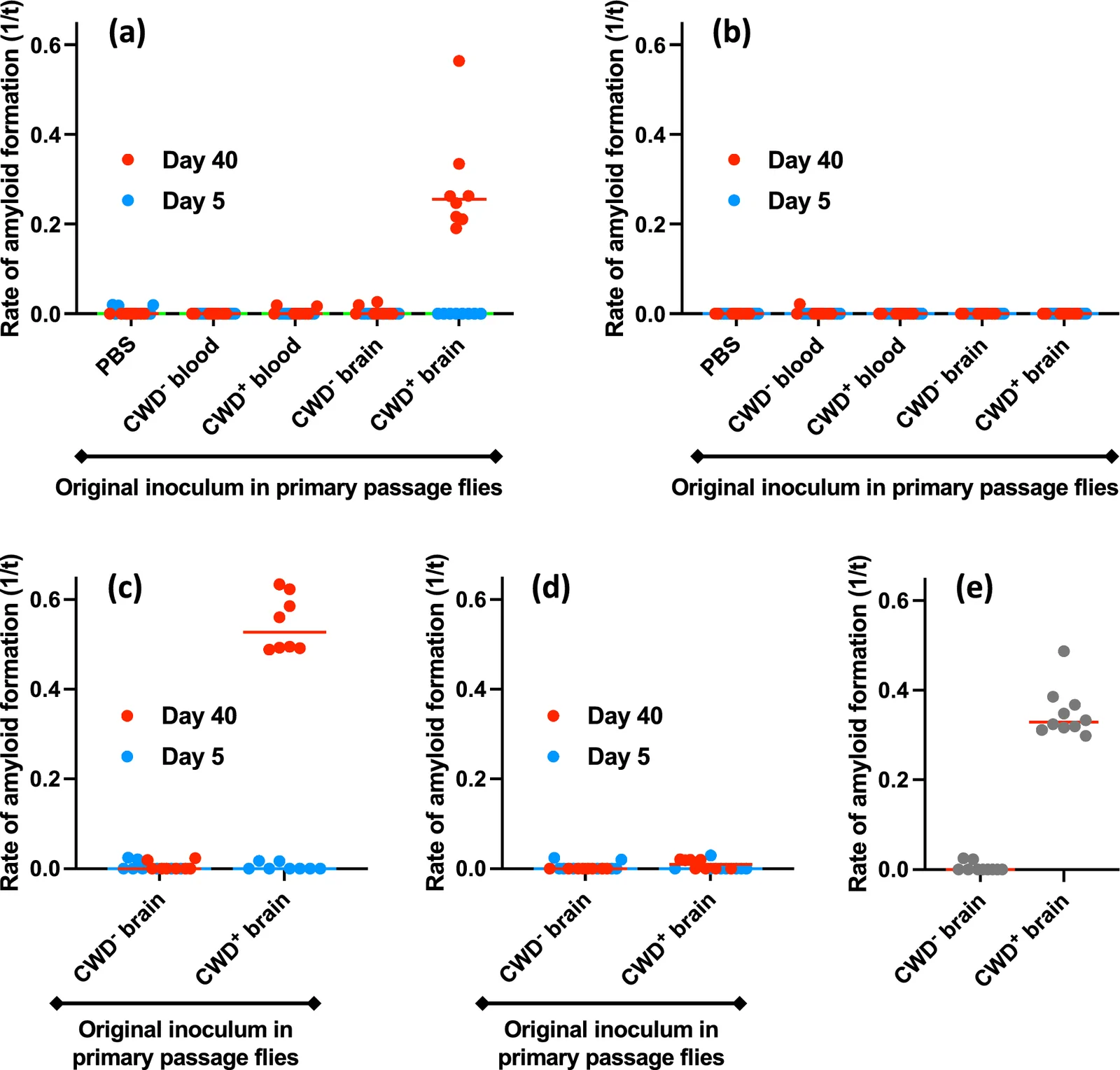
Lack of detectable prion seeding activity in cervid PrP *Drosophila* after serial passage of CWD-infected blood. Cervid whole blood and brain homogenate was passaged in *Elav* x cervid PrP *Drosophila* or *Elav* x 51D *Drosophila* (***primary*** ***passage***). The inocula at primary passage were the following: whole blood from a prion-free white-tailed deer; whole blood from a white-tailed deer with clinical experimental CWD; brain homogenate from a prion-free white-tailed deer; CWD-positive white-tailed deer brain homogenate (CBP6), or PBS. Head homogenate was prepared from 40-day old primary passage *Drosophila* and subsequently used to inoculate a fresh batch of *Elav* x cervid PrP *Drosophila* (***secondary*** ***passage***). RT-QuIC was performed on head homogenate prepared from 5- or 40-day old ***secondary passage*** *Elav* x cervid PrP *Drosophila* after primary passage in *Elav* x cervid PrP *Drosophila* (**a**) or *Elav* x 51D *Drosophila* (**b**). For experimental controls, RT-QuIC was performed on head homogenate prepared from 5- or 40-day old ***primary*** ***passage*** *Elav* x cervid PrP *Drosophila* (**c**) or *Elav* x 51D *Drosophila* (**d**). Brain homogenate from a prion-free white-tailed deer or a CWD-positive brain homogenate (CBP6) was included as RT-QuIC assay controls (**e**). In all cases the data shown are rate of amyloid formation (1/t) for each treatment group. Statistical analysis of Fig. 10 is shown in Supplementary Data 14. *Abbreviations:* CWD^**−**^ blood: whole blood from a prion-free white-tailed deer; CWD^**+**^ blood: whole blood from a white-tailed deer with clinical experimental CWD; CWD^**−**^ brain: brain homogenate from a prion-free white-tailed deer; CWD^**+**^ brain: CWD-positive brain homogenate (CBP6) from a white-tailed deer with clinical experimental CWD; PBS: phosphate buffered saline. The X axes on (**a**), (**b**), (**c**) and (**d**) are labelled to demonstrate the original inoculum in the primary passage flies that was then used to inoculate the secondary passage flies that gave rise to the RT-QuIC data shown in Fig. 10(**a**)–(**d**) inclusive

The data in Fig. 10 show that prion seeding activity was detected only in secondary passage cervid PrP *Drosophila* exposed to head homogenate from primary passage cervid PrP *Drosophila* that had been exposed to CWD-infected cervid brain homogenate (Fig. 10a). In contrast, no prion seeding activity was detected in secondary passage cervid PrP *Drosophila* exposed to head homogenate from similarly treated control 51D *Drosophila* (Fig. 10b). Transmission controls consisted of primary passage cervid PrP *Drosophila* (Fig. 10c) and control 51D *Drosophila* (Fig. 10d) exposed to CWD-infected or prion-free cervid brain material. RT-QuIC assay controls (Fig. 10e) were known CWD-infected or prion-free cervid brain material. Statistical analysis of Fig. 10 is shown in Supplementary Data 14.

## Discussion

CWD is a highly contagious prion condition that is efficiently transmitted in free-ranging and farmed cervids [5, 43, 70]. The development of an ante-mortem blood test for CWD would allow for early detection of the condition in live animals. Early detection would make a significant contribution to control measures for CWD in cervid populations and to help lower the potential risk of inter-species exposure of non-cervid hosts including humans. In this report we have successfully used cervid PrP transgenic *Drosophila* to bioassay whole blood and fractionated blood products isolated from cervids with experimental or natural pre-clinical or clinical CWD. Our observations show that cervid PrP *Drosophila* can act as a new, tractable and efficient bioassay for the sensitive detection of prion-infected blood from CWD-affected cervids.

We have previously established that cervid PrP *Drosophila* develop a neurotoxic phenotype when exposed to CWD-affected cervid brain homogenate, a response that is transmissible to cervid PrP transgenic mice [61]. This demonstrates that pan neuronal expression of cervid PrP in *Drosophila* renders this invertebrate host permissive for authentic cervid prion replication. Here we showed that cervid PrP *Drosophila* were highly susceptible to brain-derived cervid prions and were responsive to CWD-affected cervid brain inoculum diluted ≥ 10^–10^ when assessed by in vivo phenotypic bioassay or by in vitro detection of prion seeding activity. The high level of sensitivity shown by cervid PrP *Drosophila* for brain-derived cervid prions suggested this novel host could detect the perceived low levels of blood-borne CWD prions [9, 34, 45]. This appeared to be the case since we successfully demonstrated that cervid PrP *Drosophila* developed a neurotoxic phenotype after exposure to whole blood and fractionated blood products, including buffy coat cells and plasma, from CWD-affected cervids. Significantly, we showed that cervid PrP *Drosophila* developed neurotoxicity in response to blood samples collected from cervids with pre-clinical CWD. This neurotoxic response was enhanced when cervid PrP *Drosophila* were exposed to blood samples collected from donor cervids during the clinical phase of CWD. Importantly, cervid PrP *Drosophila* showed no phenotypic response after exposure to blood samples from control CWD-negative cervids. Furthermore, blood samples from CWD-affected cervids did not induce toxicity in non-PrP transgenic control 51D *Drosophila*. These data show that the neurotoxic response in cervid PrP *Drosophila* was mediated by a PrP-dependent neurotoxic moiety that accumulates in the blood of donor animals during the time course of the CWD disease. In mammalian hosts, prion-mediated neurotoxicity occurs as a consequence of prion replication [11, 36, 53] and a cardinal feature of mammalian prions is their transmissibility evidenced by serial passage between hosts [48]. Here, we successfully demonstrated the serial transmission of the CWD blood-induced neurotoxicity between cohorts of cervid PrP *Drosophila*. Collectively, these observations are consistent with the view that cervid PrP *Drosophila* are sensitive to CWD blood-borne prions. Formal proof of this view would be provided by the demonstration of accumulation of *bona fide* infectious prions or prion seeding in cervid PrP *Drosophila* previously exposed to blood from CWD-affected cervids.

We found that blood from CWD-affected cervids harbored significant levels of neurotoxicity that was distributed between cellular components and plasma, which was consistent with findings of a prionemia reported in other experimental systems [10, 28]. Buffy coat cells from CWD-affected cervids induced a neurotoxic response in cervid PrP *Drosophila* and, although not formally quantified, did so at a level comparable to that induced by cervid brain tissue-derived CWD prions. The level of the neurotoxic response decreased with decreasing numbers of buffy coat cells, and strikingly, as few as 100 buffy coat cells could induce toxicity in cervid PrP *Drosophila*. Transfusion studies in cervids have shown that blood-borne CWD prion infectivity is associated with B cells and platelets [38]. We have shown here by bioassay in cervid PrP *Drosophila* that in addition to B cells, other cervid leukocyte populations, including T cells, macrophages and neutrophils can harbor a blood-borne CWD-induced neurotoxic moiety. The difference between our data reported here and those studies performed in the natural host [38] may reflect variation in the capacity of different leukocyte subsets to harbor prion infectivity and their ability to transmit CWD disease by the intravenous route. This potential variation is supported by observations that show purified populations of blood B cells, T cells (CD4^**+**^ / CD8^**+**^) and monocytes / macrophages from classical scrapie-affected sheep can harbor prion infectivity when assessed by bioassay in ovine PrP transgenic mice but only B cells and T cells were capable of disease transmission following intravenous administration into recipient scrapie-free sheep [17, 35]. The reason for the lack of correlation between the ability of blood cells to harbor prion infectivity and their ability to induce disease transmission by the intravenous route remains unknown. It is established that the lymphoreticular system plays a fundamental role in prion neuroinvasion following peripheral prion inoculation [1, 2, 49]. Therefore, it is likely that following intravenous inoculation, cell types such as lymphocytes have a greater capacity to correctly traffic to peripheral lymphoid tissue as these cells normally re-circulate through these sites via blood and the lymphatics. An important unresolved question is the mechanism by which blood cells harbor prion infectivity. Peripheral blood cells express PrPC albeit at a relatively lower level compared to neurons of the peripheral and central nervous system, and follicular dendritic cells (FDCs) located in peripheral tissue germinal centers. Neurons and FDCs are the principal cellular locations for prion replication in mammalian hosts and are significantly more long-lived cells compared to most blood cells. Accordingly, it remains to be established whether blood cells can support prion replication or merely function as passive vehicles of prion transport. Several molecules expressed by blood mononuclear cells have been implicated in prion pathogenesis including CD21 / CD35 [29, 31].

Plasma from prion-infected hosts has been reported to contain prion infectivity although the relative capacity of this blood fraction to harbor prion infectivity compared to other blood components remains unclear. For example, plasma from CWD-affected cervids did not induce prion disease following intravenous transfusion into naïve deer or intracerebral inoculation into cervid PrP transgenic mice [38]. In addition, plasma from classical scrapie-infected sheep was less efficient than either whole blood or white blood cells in transmission of the disease when recipient scrapie-free sheep were inoculated with these samples by the intravenous route [35]. Here we showed that blood plasma from CWD-affected cervids induced neurotoxicity in cervid PrP *Drosophila* comparable to that induced by cervid whole blood. This suggests that the level of prion infectivity in blood plasma of prion diseased individuals may be greater than that previously considered. Our previous observations have shown that blood plasma from classical scrapie-infected sheep was neurotoxic in ovine PrP transgenic *Drosophila* after dilution by multiple orders of magnitude [57], but appeared to contain a low level of infectivity when assayed in the natural host [35]. The nature of prion infectivity in blood plasma remains unknown. In vitro binding studies have shown that brain-derived PrPSc can complex with apolipoprotein B, a major component of very low-density lipoproteins (VLDL) and low-density lipoproteins (LDL), which suggests VLDLs and LDLs transport prions in blood plasma [52]. In addition, PrPSc has been detected in blood plasma associated with exosomes [50], which are extracellular membrane vesicles secreted by most cells after fusion of multivesicular bodies with the plasma membrane [18]. These observations suggest that isolation of specific blood components harboring prions from the plasma of CWD-affected cervids could provide the basis of new diagnostic tests for prion disease in these animals. For example, plasminogen, which has been reported to bind PrPSc [21], has been used to detect disease-associated PrP in plasma [56].

We have demonstrated that whole blood and blood fractions from CWD-affected cervids induce a neurotoxic phenotype in cervid PrP *Drosophila*, in a manner considered to be compatible with prion detection. Significantly, this blood-borne prion-induced neurotoxic phenotype was not associated with the accumulation of detectable prion seeding activity in cervid PrP *Drosophila*, unlike the response induced by brain-derived CWD prions. However, we have demonstrated that cervid PrP *Drosophila* were able to accumulate a transmissible toxicity after exposure to CWD-affected cervid blood by secondary transmission of head homogenate from these flies to naïve cervid PrP *Drosophila* that subsequently developed a neurotoxic phenotype. Interestingly, despite the onset of a neurotoxic phenotype, head homogenate from these second passage cervid PrP *Drosophila* failed to accumulate detectable prion seeding activity, which was also not detectable in CWD-affected blood exposed primary passage flies. In a similar manner, we have previously demonstrated that ovine PrP *Drosophila* exposed to blood plasma from classical scrapie-affected sheep developed a neurotoxic phenotype but brain homogenate prepared from these flies did not routinely show evidence of PK-resistant PrPSc by PMCA and did not induce clinical prion disease when inoculated into tg338 ovine PrP transgenic mice [57]. Accordingly, if our view is correct that cervid PrP *Drosophila* can detect *bona fide* blood-borne cervid prions, we propose that mammalian blood-borne and brain-derived prions have distinct propagation properties in PrP transgenic *Drosophila*. This may arise because different prion repertoires exist within these two body compartments. For example, while mammalian brain tissue and blood may contain a common prion conformer responsible for the transmissible neurotoxic phenotype in PrP transgenic *Drosophila*, brain tissue may contain a prion conformer that mediates accumulation of in vitro detectable prion seeding activity, one that is either absent or present at a sufficiently lower level in blood so as not to be detected by PrP transgenic *Drosophila*. In one extreme, the relatedness between these proposed different prion conformers could represent different prion strains. In support of these proposals are the hypotheses that transmissible prions comprise an ensemble of PrPSc conformers [13] and that the neurotoxic prion moiety may comprise PK-sensitive or PK-resistant PrPSc [51], and the observations that brain tissue can harbor different prion strains compared to peripheral lymphoid tissue [6, 16]. Whatever the case, the suggested high sensitivity shown by cervid PrP *Drosophila* for blood-borne CWD prions compared to the natural cervid host may arise because of the unique manner of mammalian PrP expression in *Drosophila*. In support of this view, we have recently shown that PrP transgenic *Drosophila* either lack or have an altered transmission barrier for mammalian prions compared to vertebrate species [62].

## Conclusions

In summary, we have provided good evidence to support the view that cervid PrP *Drosophila* can readily detect cervid prions in whole blood, different blood cell populations and in plasma from CWD-affected cervids. Significantly, we have found that cervid PrP *Drosophila* are capable of the detection of whole blood and plasma from cervids with pre-clinical CWD. The development of an ante-mortem cervid blood test for CWD would represent an important step in the control and management of this highly contagious prion disease. Collectively, our findings highlight a role for cervid PrP transgenic *Drosophila* in the development of a confirmatory blood test for prion infectivity in prion-diseased cervids.

## Supplementary Information

Supplementary Material 1.

Supplementary Material 2.

## Data Availability

All data generated or analysed during this study are included in the published article [and its supplementary information files].

## Abbreviations

BSE: Bovine spongiform encephalopathy
CJD: Creutzfeldt-Jakob disease
CWD: Chronic wasting disease
FDCs: Follicular dendritic cells
LDL: Low-density lipoproteins
NBH: Normal brain homogenate
PBMC: Peripheral blood mononuclear cells
PBS: Phosphate buffered saline
PK: Proteinase K
PMCA: Protein misfolding cyclic amplification
PrP: Prion protein
PrPC: Normal cellular PrP
PrPSc: Abnormal disease-specific conformation of PrP
RT-QuIC: Real-time quaking-induced conversion
SDS-PAGE: Sodium dodecyl-sulphate polyacrylamide gel electrophoresis
ThT: Thioflavin T
VLDL: Very low-density lipoproteins
