# Supplementary material for: Successful bioassay of blood from chronic wasting disease-affected cervids in cervid PrP transgenic Drosophila
Source: Acta Neuropathol Commun. 2026 Jul 15;14:211. doi: 10.1186/s40478-026-02372-1 (PMC13651417; doi:10.1186/s40478-026-02372-1)
Supplement: Supplementary file 1 [file 40478_2026_2372_MOESM1_ESM.pdf]

Article Title: Successful bioassay of blood from chronic wasting disease-affected cervids in cervid PrP transgenic *Drosophila*

Journal Name: Acta Neuropathologica Communications

Author names: Alana M. Thackray, Erin E. McNulty, Amy V. Nalls, Marcelo H. Jorge, Michael J. Chamberlain, Mark G. Ruder, Candace K. Mathiason and Raymond Bujdoso

Corresponding author name: Raymond Bujdoso

Corresponding author email: rb202@cam.ac.uk

Corresponding author affiliation: University of Cambridge, Department of Veterinary Medicine, Madingley Road, Cambridge, CB3 0ES, UK

Supplementary Data 1 Summary table of cervid inocula

Figure number	Cervid identification	Sample	Dilution used	Study cohort	CWD status and clinical presentation
Figure 2 and Supplementary Data 2	CBP6	Brain homogenate	Dilution series of 10^{-2} to 10^{-18}	Experimental	CWD positive (brain pool consisting of IHC positive brain sections from 6 experimental CWD-inoculated WTD)
	#502/504	Normal brain homogenate	10^{-2}	Experimental	CWD negative (brain pool consisting of the same sections as above from 2 negative control WTD; IHC negative at termination)
Figure 3 and Supplementary Data 3	#1308	Whole blood	10^{-2}	Experimental	IHC positive, terminal CWD sample collected at study termination
	#1308	Plasma	10^{-2}	Experimental	IHC positive, terminal CWD sample collected at study termination
	#1316	Whole blood	10^{-2}	Experimental	IHC positive, terminal CWD sample collected at study termination
	#1316	Plasma	10^{-2}	Experimental	IHC positive, terminal CWD sample collected at study termination
	#1437	Whole blood	10^{-2}	Experimental	IHC negative, control WTD, no clinical signs, sample collected at study termination
	#1437	Plasma	10^{-2}	Experimental	IHC negative, control WTD, no clinical signs, sample collected at study termination
Figure 4 and Supplementary Data 4	#1308	Buffy coat	10^{2}, 10^{4}, 10^{6} cells	Experimental	IHC positive, terminal CWD sample collected at study termination
	#1316	Buffy coat	10^{2}, 10^{4}, 10^{6} cells	Experimental	IHC positive, terminal CWD sample collected at study termination
	#1437	Buffy coat	10^{2}, 10^{4}, 10^{6} cells	Experimental	IHC negative, control WTD, no clinical signs, sample collected at study termination
Figure 5 and Supplementary Data 5	#1528	PBMC	10^{5} cells	Experimental	CWD positive, sample collected at terminal clinical disease
	#1528	Buffy coat	10^{5} cells	Experimental	CWD positive, sample collected at terminal clinical disease
	#1528	CD4+ cells	10^{5} cells	Experimental	CWD positive, sample collected at terminal clinical disease
	#1697	CD8+ cells	10^{5} cells	Experimental	CWD positive, sample collected at terminal clinical disease
	#1528	CD14+ cells	10^{5} cells	Experimental	CWD positive, sample collected at terminal clinical disease

**Figure number**	**Cervid identification**	**Sample**	**Dilution used**	**Study cohort**	**CWD status and clinical presentation**
Figure 5 and Supplementary Data 5	#1528	B cells	10^5 cells	Experimental	CWD positive, sample collected at terminal clinical disease
	#1528	Neutrophils	10^5 cells	Experimental	CWD positive, sample collected at terminal clinical disease
	#1528	Platelets	10^5 cells	Experimental	CWD positive, sample collected at terminal clinical disease
	#1531	PBMC	10^5 cells	Experimental	CWD negative, sample collected at study termination
	#1531	Buffy coat	10^5 cells	Experimental	CWD negative, sample collected at study termination
	#1531	CD4+ cells	10^5 cells	Experimental	CWD negative, sample collected at study termination
	#1531	CD8+ cells	10^5 cells	Experimental	CWD negative, sample collected at study termination
	#1531	CD14+ cells	10^5 cells	Experimental	CWD negative, sample collected at study termination
	#1531	B cells	10^5 cells	Experimental	CWD negative, sample collected at study termination
	#1531	Neutrophils	10^5 cells	Experimental	CWD negative, sample collected at study termination
	#1531	Platelets	10^5 cells	Experimental	CWD negative, sample collected at study termination
Supplementary Data 6a and 7	#1697	Whole blood	10^{-2}	Experimental	IHC positive, sample collected at terminal clinical disease
	#1697	Platelet-rich plasma	10^{-2}	Experimental	IHC positive, sample collected at terminal clinical disease
	#1697	Cell-free plasma	10^{-2}	Experimental	IHC positive, sample collected at terminal clinical disease
Supplementary Data 6b and 7	#1531	Whole blood	10^{-2}	Experimental	CWD negative, sample collected at study termination
	#1531	Platelet-rich plasma	10^{-2}	Experimental	CWD negative, sample collected at study termination
	#1531	Cell-free plasma	10^{-2}	Experimental	CWD negative, sample collected at study termination
Figure 6 and Supplementary Data 8	#906-1	Whole blood	10^{-2}	Natural	IHC positive, (deer was determined lymphoid biopsy positive at sample collection point but appeared healthy)
	#906-10	Whole blood	10^{-2}	Natural	IHC positive, (deer was determined lymphoid biopsy positive at sample collection point but appeared healthy)
	#906-2	Whole blood	10^{-2}	Natural	CWD positive, (deer was determined lymphoid biopsy positive at sample collection point but appeared healthy)

**Figure number**	**Cervid identification**	**Sample**	**Dilution used**	**Study cohort**	**CWD status and clinical presentation**
Figure 6 and Supplementary Data 8	#906-6	Whole blood	10^{-2}	Natural	CWD positive, (deer was determined lymphoid biopsy positive at sample collection point but appeared healthy)
	#904-1	Whole blood	10^{-2}	Natural	IHC negative, (deer was determined lymphoid biopsy negative at sample collection point and appeared healthy)
	#904-2	Whole blood	10^{-2}	Natural	IHC negative, (deer was determined lymphoid biopsy negative at sample collection point and appeared healthy)
Figure 7 and Supplementary Data 9	#1308	Whole blood (3 months P.I.)	10^{-2}	Experimental	IHC positive, (sample collected at 3 months P.I., deer was determined lymphoid biopsy positive at sample collection point but appeared healthy)
	#1308	Whole blood (6 months P.I.)	10^{-2}	Experimental	IHC positive, (sample collected at 6 months P.I., deer was determined lymphoid biopsy positive at sample collection point but appeared healthy)
	#1308	Whole blood (12 months P.I.)	10^{-2}	Experimental	IHC positive, (sample collected at 12 months P.I., deer was determined lymphoid biopsy positive at sample collection point but appeared healthy)
	#1308	Whole blood (18 months P.I.)	10^{-2}	Experimental	IHC positive, terminal CWD, (sample collected at 18 months P.I., deer was showing late stage CWD clinical disease and was determined post-mortem brain, tonsil and RAMALT positive at sample collection point)
	#1444	Whole blood (18 months P.I.)	10^{-2}	Experimental	IHC negative, control WTD, no clinical signs, sample collected at study termination
	#1308	Plasma (3 months P.I.)	10^{-2}	Experimental	IHC positive, (sample collected at 3 months P.I., deer was determined lymphoid biopsy positive at sample collection point but appeared healthy)
	#1308	Plasma (15 months P.I.)	10^{-2}	Experimental	IHC positive, (sample collected at 15 months P.I., deer was determined lymphoid biopsy positive at sample collection point and showing early signs of CWD clinical disease)
	#1316	Plasma (3 months P.I.)	10^{-2}	Experimental	IHC positive, (sample collected at 3 months P.I., deer was determined lymphoid biopsy positive at sample collection point but appeared healthy)

Figure number	Cervid identification	Sample	Dilution used	Study cohort	CWD status and clinical presentation
Figure 7 and Supplementary Data 9	#1316	Plasma (15 months P.I.)	10^{-2}	Experimental	IHC positive, (sample collected at 15 months P.I., deer was determined lymphoid biopsy positive at sample collection point and showing early signs of CWD clinical disease)
	#1444 *	Plasma (3 months P.I.)	10^{-2}	Experimental	IHC negative, control WTD, no clinical signs, sample collected at 3 month P.I.
	#1444	Plasma (15 months P.I.)	10^{-2}	Experimental	IHC negative, control WTD, no clinical signs sample collected at 15 month P.I.
Figure 8, Supplementary Data 10, 11 and 12	#906-10	Whole blood (2.75 years)	10^{-2}	Natural	IHC positive, (deer was determined lymphoid biopsy positive at sample collection point but appeared healthy)
	#269	Whole blood (2.5 years)	10^{-2}	Natural	IHC and RT-QuIC positive, (deer was determined lymphoid biopsy positive at sample collection point but appeared healthy)
	#325	Whole blood (8 months)	10^{-2}	Natural	Low IHC, RT-QuIC positive, (deer was determined lymphoid biopsy positive at sample collection point but appeared healthy)
	#904-2	Whole blood (1.75 years)	10^{-2}	Natural	IHC negative, (deer was determined lymphoid biopsy negative at sample collection point and appeared healthy)
	ACD pool	Whole blood (7 months)	10^{-2}	Experimental	CWD negative, blood pool collected from multiple CWD negative WTD
	#905-6	Buffy coat (4.75 years)	10^{6} cells	Natural	IHC positive, (deer was determined lymphoid biopsy positive at sample collection point but appeared thin)
	#237	Buffy coat (3.5 years)	10^{6} cells	Natural	IHC and RT-QuIC positive, (deer was determined lymphoid biopsy positive at sample collection point but appeared healthy)
	#906-10	Buffy coat (2.75 years)	10^{6} cells	Natural	IHC positive, (deer was determined lymphoid biopsy positive at sample collection point but appeared healthy)
	#269	Buffy coat (1.5 years)	10^{6} cells	Natural	IHC and RT-QuIC positive, (deer was determined lymphoid biopsy positive at sample collection point but appeared healthy)
	#325	Buffy coat (8 months)	10^{6} cells	Natural	Low IHC, RT-QuIC positive, (deer was determined lymphoid biopsy positive at sample collection point but appeared healthy)
	#904-2	Buffy coat (1.75 years)	10^{6} cells	Natural	IHC negative, (deer was determined lymphoid biopsy negative at sample collection point and appeared healthy)

Figure number	Cervid identification	Sample	Dilution used	Study cohort	CWD status and clinical presentation
Figure 8, Supplementary Data 10, 11 and 12	ACD pool	Buffy coat (7 months)	10^6 cells	Experimental	CWD negative, blood pool collected from multiple CWD negative WTD
	CBP6	Brain homogenate	10^{-2}	Experimental	CWD positive (brain pool consisting of IHC positive brain sections from 6 experimental CWD-inoculated WTD)
	#502/504	Normal brain homogenate	10^{-2}	Experimental	CWD negative (brain pool consisting of the same sections as above from 2 negative control WTD; IHC negative at termination)
Figure 9 and Supplementary Data 13	#1316	Whole blood	10^{-2}	Experimental	IHC positive, terminal CWD, (deer was determined lymphoid biopsy positive at sample collection point for tonsil and RAMALT)
	#1437	Whole blood	10^{-2}	Experimental	IHC negative, control WTD, no clinical signs, sample collected at study termination
	CBP6	Brain homogenate	10^{-2}	Experimental	CWD positive (brain pool consisting of IHC positive brain sections from 6 experimental CWD-inoculated WTD)
	#502/504	Normal brain homogenate	10^{-2}	Experimental	CWD negative (brain pool consisting of the same sections as above from 2 negative control WTD; IHC negative at termination)
Figure 10 and Supplementary Data 14	#1316	Whole blood	10^{-2}	Experimental	IHC positive, terminal CWD, (deer was determined lymphoid biopsy positive at sample collection point for tonsil and RAMALT)
	#1437	Whole blood	10^{-2}	Experimental	IHC negative, control WTD, no clinical signs, sample collected at study termination
	CBP6	Brain homogenate	10^{-2}	Experimental	CWD-positive (brain pool consisting of IHC positive brain sections from 6 experimental CWD-inoculated WTD)
	#502/504	Normal brain homogenate	10^{-2}	Experimental	CWD-negative (brain pool consisting of the same sections as above from 2 negative control WTD; IHC negative at termination)

Footnote:
IHC = Immunohistochemistry. All IHC testing was carried out on lymphoid tissue unless otherwise stated
WTD = White-tailed deer
# = Cervid identification number
P.I. = Post inoculation
RAMALT = Rectoanal mucosa-associated lymphoid tissue
***** Sample not used in Figure 7d

Supplementary Data 2 Statistical analysis of accelerated decline of locomotor activity and loss of survival in CWD brain homogenate-exposed cervid PrP *Drosophila*

CWD-positive cervid brain homogenate dilution	RT-QuIC	Phenotypic assay	
	Seeding activity (Expt. 2a)	Locomotor activity (Expt. 2b)	Survival (Expt. 2c)
10^{-2}	p=0.0002	p<0.0001	69* / p<0.0001
10^{-4}	p=0.0002	p<0.0001	76* / p<0.0001
10^{-6}	p=0.0002	p<0.0001	80* / p<0.0001
10^{-8}	p=0.0002	p=0.0019	85* / p<0.0001
10^{-10}	p=0.0002	p=0.0046	85* / p<0.0001
10^{-12}	p>0.9999	p=0.0335	87* / p<0.0001
10^{-14}	p>0.9999	p=0.0060	92* / p<0.0001
10^{-16}	p>0.9999	p<0.1588	94* / p=0.0067
10^{-18}	p>0.9999	p=0.0814	120* / p=0.7878

Supplementary Data 2 Legend. *Elav* x cervid PrP *Drosophila* were exposed at the larval stage to a 10^{-2} dilution series of experimentally infected CWD-positive white-tailed deer brain homogenate (CBP6), or a 10^{-2} dilution of CWD-negative cervid normal brain homogenate (NBH) (cervid #502/504), or phosphate buffered saline (PBS). After hatching, adult *Drosophila* were assessed for prion seeding activity by RT-QuIC (see Figure 2a), locomotor activity by a negative geotaxis climbing assay (see Figure 2b) or for survival (see Figure 2c). Statistical analysis of the RT-QuIC data was performed using an unpaired, non-parametric, two-tailed Mann-Whitney test. Statistical analysis of the negative geotaxis climbing assay data was performed using an unpaired (two-tailed) Student t test. The Log-rank (Mantel-Cox) test was used to assess the survival assay data. In all analyses, each prion treatment group was compared to the NBH 10^{-2} treatment group (cervid #502/504). Statistical analysis of median survival times was carried out using Kaplan-Meier plots. Comparisons that are statistically significantly different (p<0.05) are shown in red. Comparison of the NBH 10^{-2} treatment groups (cervid #502/504) with PBS were not statistically significantly different (p>0.05, data not shown). # cervid identification number; * = median survival time in days of prion treatment group.

Supplementary Data 3 Statistical analysis of accelerated decline of locomotor activity and loss of survival in experimental CWD-infected whole blood or plasma-exposed cervid PrP *Drosophila*

Inoculum	Cervid PrP *Drosophila*		51D *Drosophila*	
	Locomotor activity (Expt. 3a)	Survival (Expt. 3b)	Locomotor activity (Expt. 3c)	Survival (Expt. 3d)
**Whole blood CWD-positive (#1308)**	***p*=0.0103**	**64* / *p*<0.0001**	***p*=0.6711**	**99* / *p*=0.5530**
**Whole blood CWD-positive (#1316)**	***p*=0.0338**	**52* / *p*<0.0001**	***p*=0.7058**	**104* / *p*=0.6851**
**Plasma CWD-positive (#1308)**	***p*=0.0012**	**52* / *p*<0.0001**	***p*=0.8141**	**101* / *p*=0.6705**
**Plasma CWD-positive (#1316)**	***p*=0.0019**	**55* / *p*<0.0001**	***p*=0.6148**	**104* / *p*=0.2696**

Supplementary Data 3 Legend. *Elav* x cervid PrP *Drosophila* or *Elav* x 51D *Drosophila* were exposed at the larval stage to a 10^{-2} dilution of whole blood or plasma isolated from clinical experimentally infected CWD-positive or CWD-negative white-tailed deer, or PBS. After hatching, adult *Drosophila* were assessed for locomotor activity by a negative geotaxis climbing assay (see Figure 3a and 3c) or for survival (see Figure 3b and 3d). Statistical analysis of the negative geotaxis climbing assay data was performed using an unpaired (two-tailed) Student t test. The Log-rank (Mantel-Cox) test was used to assess the survival assay data. In both analyses, each CWD-positive treatment group was compared to its respective CWD-negative treatment group, which were from cervid #1437. Statistical analysis of median survival times was carried out using Kaplan-Meier plots. Comparisons that are statistically significantly different (p<0.05) are shown in red. Comparison of WB CWD-negative or PL CWD-negative treatment groups with PBS were not statistically significantly different (p>0.05, data not shown) except for PBS vs whole blood CWD-negative survival assay data *(p*=0.0203). # = cervid identification number; * = median survival time in days of prion treatment group. Abbreviations: WB = whole blood; PL = plasma; PBS = phosphate buffered saline.

Supplementary Data 4 Statistical analysis of accelerated decline of locomotor activity and loss of survival in experimental CWD-infected buffy coat cell-exposed cervid PrP *Drosophila*

	Cervid PrP *Drosophila*		51D *Drosophila*	
Inoculum	Locomotor activity (Expt. 4a)	Survival (Expt. 4b)	Locomotor activity (Expt. 4c)	Survival (Expt. 4d)
10^6 CWD-positive cells (#1308)	p=0.0008	52* / p<0.0001	p=0.4983	101* / p=0.5329
10^4 CWD-positive cells (#1308)	p=0.0055	57* / p<0.0001	p=0.8958	104* / p=0.6911
10^2 CWD-positive cells (#1308)	p=0.0736	52* / p<0.0001	P=0.4473	106* / p=0.5678
10^6 CWD-positive cells (#1316)	p=0.0006	50* / p<0.0001	p=0.4691	106* / p=0.5979
10^4 CWD-positive cells (#1316)	p=0.0033	50* / p<0.0001	p=0.9226	106* / p=0.4935
10^2 CWD-positive cells (#1316)	p=0.0207	52* / p<0.0001	p=0.7012	108* / p=0.9914

Supplementary Data 4 Legend. *Elav* x cervid PrP *Drosophila* or *Elav* x 51D *Drosophila* were exposed at the larval stage to 10^2, 10^4 or 10^6 buffy coat cells from clinical experimentally infected CWD-positive or CWD-negative white-tailed deer, or phosphate buffered saline (PBS). After hatching, adult *Drosophila* were assessed for locomotor activity by a negative geotaxis climbing assay (see Figure 4a and 4c) or for survival (see Figure 4b and 4d). Statistical analysis of the negative geotaxis climbing assay data was performed using an unpaired (two-tailed) Student t test. The Log-rank (Mantel-Cox) test was used to assess the survival assay data. In both analyses, each CWD-positive treatment group was compared to its respective CWD-negative treatment group, which were from cervid #1437. Statistical analysis of median survival times was carried out using Kaplan-Meier plots. Comparisons that are statistically significantly different (p<0.05) are shown in red. Comparison of CWD-negative buffy coat cell treatment groups with PBS were not statistically significantly different (p>0.05, data not shown). # = cervid identification number; * = median survival time in days of prion treatment group.

Supplementary Data 5 Statistical analysis of accelerated decline of locomotor activity in CWD-infected blood leukocyte subset-exposed cervid PrP *Drosophila*

Inoculum	Locomotor activity (Expt. 5)
PBMC	$p<0.0001$
Buffy coat cells	$p<0.0001$
$CD4^+$ T cells	$p<0.0001$
$CD8^+$ T cells	$p<0.0001$
$CD14^+$ macrophages	$p<0.0001$
B cells	$p<0.0001$
Neutrophils	$p<0.0001$
Platelets	$p<0.0001$

Supplementary Data 5 Legend. Adult *Elav* x cervid PrP *Drosophila* were assessed for locomotor activity by a negative geotaxis climbing assay after exposure at the larval stage to homogenate prepared from 10^5 cells of various blood leukocyte subsets isolated from clinical experimentally infected CWD-positive or CWD-negative white-tailed deer, or PBS (see Figure 5). Statistical analysis of the negative geotaxis climbing assay data was performed using an unpaired (two-tailed) Student t test. CWD-positive blood leukocyte subset treatment groups (all from cervid #1528, except $CD8^+$ cells that were from cervid #1697) were compared to their respective CWD-negative blood leukocyte subset treatment group (all from cervid #1531). Comparisons that are statistically significantly different ($p<0.05$) are shown in red. Comparison of CWD-negative blood leukocyte subset treatment groups (all from cervid #1531) with PBS were not statistically significantly different ($p>0.05$, data not shown) except for the negative PBMC, $CD4^+$ T cells and platelets compared to PBS ($p=0.0425$, $p=0.0450$ and $p=0.0345$ respectively).
Abbreviation: PBMC = peripheral blood mononuclear cells; PBS = phosphate buffered saline.

Supplementary Data 6 Accelerated decline of locomotor activity in CWD-infected plasma-exposed cervid PrP *Drosophila*

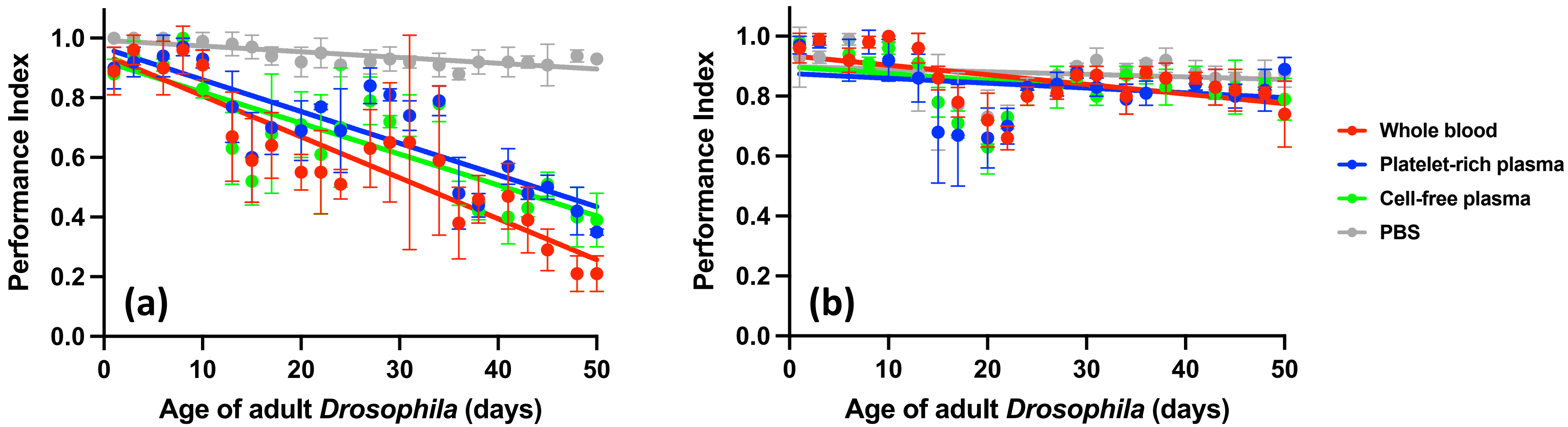

Supplementary Data 6 Legend. Adult *Elav* x cervid PrP *Drosophila* were assessed for locomotor activity by a negative geotaxis climbing assay after exposure at the larval stage to a 10^{-2} dilution of whole blood; platelet-rich plasma; cell-free plasma from (a) clinical experimentally infected CWD-positive (cervid #1697) or (b) CWD-negative (cervid #1531) white-tailed deer, or phosphate buffered saline (PBS) (a) and (b). The mean performance index (± SD), expressed as a regression line, is shown for three groups of n=15 flies per time point. Statistical analysis of Supplementary Data 6 is shown in Supplementary Data 7.

Supplementary Data 7 Statistical analysis of accelerated decline of locomotor activity in CWD-infected plasma-exposed cervid PrP *Drosophila*

Inoculum	Locomotor activity (Expt. Supplementary Data 6)
**Whole blood**	***p*<0.0001**
**Platelet-rich plasma**	***p*=0.0036**
**Cell-free plasma**	***p*<0.0002**

Supplementary Data 7 Legend. Adult *Elav* x cervid PrP *Drosophila* were assessed for locomotor activity by a negative geotaxis climbing assay after exposure at the larval stage to a 10^{-2} dilution of whole blood; platelet-rich plasma; cell-free plasma from a clinical experimentally infected CWD-positive (cervid #1697, see Supplementary Data 6a) or a CWD-negative white-tailed deer (cervid #1531, see Supplementary Data 6b), or phosphate buffered saline (PBS). Statistical analysis of the negative geotaxis climbing assay data was performed using an unpaired (two-tailed) Student t test with CWD-positive treatment groups compared to their respective CWD-negative treatment group. Comparisons that were statistically significantly different ($p<0.05$) are shown in red. Comparison of CWD-negative whole blood, platelet-rich plasma or cell-free plasma treatment groups (cervid #1531) with PBS were not statistically significantly different ($p>0.05$, data not shown). # = cervid identification number.

Supplementary Data 8 Statistical analysis of accelerated decline of locomotor activity and loss of survival in natural CWD-infected whole blood-exposed cervid PrP *Drosophila*

	Cervid PrP *Drosophila*		51D *Drosophila*	
**Inoculum**	**Locomotor activity (Expt. 6a)**	**Survival (Expt. 6b)**	**Locomotor activity (Expt. 6c)**	**Survival (Expt. 6d)**
**Whole blood CWD-positive (#906-1)**	***p*=0.0023**	**55* / *p*<0.0001**	***p*=0.9031**	**106* / *p*=0.5442**
**Whole blood CWD-positive (#906-10)**	***p*=0.0014**	**55* / *p*<0.0001**	***p*=0.8650**	**104* / *p*=0.7577**
**Whole blood CWD-positive (#906-2)**	***p*=0.0020**	**52* / *p*<0.0001**	***p*=0.5186**	**106* / *p*=0.5684**
**Whole blood CWD-positive (#906-6)**	***p*=0.0019**	**55* / *p*<0.0001**	***p*=0.6753**	**104* / *p*=0.8377**

Supplementary Data 8 Legend. *Elav* x cervid PrP *Drosophila* or *Elav* x 51D *Drosophila* were exposed at the larval stage to a 10^{-2} dilution of whole blood from natural CWD exposed (CWD-positive) or from prion-free (CWD-negative) white-tailed deer, or phosphate buffered saline (PBS). After hatching, adult *Drosophila* were assessed for locomotor activity by a negative geotaxis climbing assay (see Figure 6a and 6c) or for survival (see Figure 6b or 6d). Statistical analysis of the negative geotaxis climbing assay data was performed using an unpaired (two-tailed) Student t test. The Log-rank (Mantel-Cox) test was used to assess the survival assay data. In both analyses, each CWD-positive whole blood treatment group was compared to the CWD-negative whole blood sample from cervid #904-1. Statistical analysis of median survival times was carried out using Kaplan-Meier plots. Comparisons that are statistically significantly different ($p<0.05$) are shown in red. Comparison between CWD-negative whole blood treatment group data from cervids #904-1 with #904-2, or both of these treatment groups individually with PBS were not statistically significantly different ($p>0.05$, data not shown). # = cervid identification number; * = median survival time in days of prion treatment group.

Supplementary Data 9 Statistical analysis of accelerated decline of locomotor activity induced by pre-clinical experimental CWD time-course blood and plasma sample-exposed cervid PrP *Drosophila*

	Locomotor activity	
**Whole blood inoculum**	**Cervid PrP *Drosophila* (Expt. 7a)**	**51D *Drosophila* (Expt. 7b)**
**18 months P.I. CWD-positive (#1308)**	***p*<0.0001**	***p*=0.8863**
**12 months P.I. CWD-positive (#1308)**	***p*<0.0001**	***p*=0.8804**
**6 months P.I. CWD-positive (#1308)**	***p*<0.0001**	***p*=0.7974**
**3 months P.I. CWD-positive (#1308)**	***p*<0.0001**	***p*=0.4675**
**Plasma inoculum**	**Cervid PrP *Drosophila* (Expt. 7c)**	**51D *Drosophila* (Expt. 7d)**
**15 months P.I. CWD-positive (#1308)**	***p*<0.0001**	***p*=0.3249**
**3 months P.I. CWD-positive (#1308)**	***p*<0.0001**	***p*=0.2898**
**15 months P.I. CWD-positive (#1316)**	***p*<0.0001**	***p*=0.5207**
**3 months P.I. CWD-positive (#1316)**	***p*=0.0032**	***p*=0.3696**

Supplementary Data 9 Legend. *Elav* x cervid PrP *Drosophila* or *Elav* x 51D *Drosophila* were exposed at the larval stage to a 10^{-2} dilution of whole blood or plasma time course samples isolated from experimentally infected (CWD-positive) or prion-free (CWD-negative) white-tailed deer, or PBS. After hatching, adult *Drosophila* were assessed for locomotor activity by a negative geotaxis climbing assay (see Figure 7). Statistical analysis of the negative geotaxis climbing assay data was performed using an unpaired (two-tailed) Student t test. CWD-positive whole blood treatment groups were compared to an 18 months old CWD-negative whole blood treatment group (cervid #1444). CWD-positive plasma treatment groups were compared to a 15 months old CWD-negative plasma treatment group (cervid #1444). Comparisons that are statistically significantly different ($p<0.05$) are shown in red. Comparison between CWD-negative whole blood (18 months old cervid #1444) with PBS or CWD-negative plasma treatment group data from cervid #1444 (3 months and 15 months old), or both of these plasma treatment groups individually with PBS were not statistically significantly different ($p>0.05$, data not shown).
# = cervid identification number. Abbreviations: P.I. = post inoculation; PBS = phosphate buffered saline.

Supplementary Data 10 Statistical analysis of accelerated decline of locomotor activity and loss of survival in defined age natural CWD-infected whole blood or buffy coat sample-exposed cervid PrP *Drosophila*

	Whole Blood		Buffy Coat	
**Inoculum**	**Locomotor activity (Expt. 8a)**	**Survival (Expt. 8b)**	**Locomotor activity (Expt. 8c)**	**Survival (Expt. 8d)**
**8 months old CWD-positive (#325)**	p=0.0007	71* / p<0.0001	p=0.0015	85* / p<0.0001
**1.5 years old CWD-positive (#269)**	N/A	N/A	p=0.0016	80* / p<0.0001
**2.5 years old CWD-positive (#269)**	p=0.0003	71* / p<0.0001	N/A	N/A
**2.75 years old CWD-positive (#906-10)**	p=0.0013	66* / p<0.0001	p=0.0003	69* / p<0.0001
**3.5 years old CWD-positive (#237)**	N/A	N/A	p=0.0004	59* / p<0.0001
**4.75 years old CWD-positive (#905-6)**	N/A	N/A	p=0.0002	55* / p<0.0001
**CWD-positive brain (CBP6)**	p<0.0001	57* / p<0.0001	p<0.0001	57* / p<0.0001

Supplementary Data 10 Legend. *Elav* x cervid PrP *Drosophila* were exposed at the larval stage to a 10^{-2} dilution of defined age samples of whole blood or 10^{6} buffy coat cells from cervids with natural exposure to CWD (CWD-positive) or CWD-negative white-tailed deer, or a 10^{-2} dilution of CWD-positive cervid brain homogenate (CBP6), or a 10^{-2} dilution of CWD-negative cervid brain homogenate (cervid #502/504), or phosphate buffered saline (PBS). After hatching, adult *Drosophila* were assessed for locomotor activity by a negative geotaxis climbing assay (see Figure 8a and 8c) or for survival (see Figure 8b and 8d). Statistical analysis of the negative geotaxis climbing assay data was performed using an unpaired (two-tailed) Student t test. The Log-rank (Mantel-Cox) test was used to assess the survival assay data. In both analyses, each CWD-positive treatment group was compared to the 1.75 years old CWD-negative treatment group (cervid #904-2). The CBP6 CWD-positive brain homogenate treatment group was compared to the CWD-negative brain homogenate treatment group (cervid #502/504). Statistical analysis of median survival times was carried out using Kaplan-Meier plots. Comparisons that are statistically significantly different (p<0.05) are shown in red. Comparison of CWD-negative treatment groups with PBS were not statistically significantly different (p>0.05, data not shown) except the whole blood and buffy coat survival data analysis of the 1.75 years old CWD-negative sample (cervid #904-2) (p=0.0013 and p=0.0014 respectively) and the whole blood and buffy coat survival data analysis of the 7 months old CWD-negative sample (ACD pool) (p=0.0011 and p=0.0008 respectively). # = cervid identification number. * = median survival time in days of prion treatment group; N/A = not applicable.

Supplementary Data 11 Lack of accelerated loss of locomotor activity and survival in defined age natural CWD-infected whole blood or buffy coat sample-exposed 51D *Drosophila*

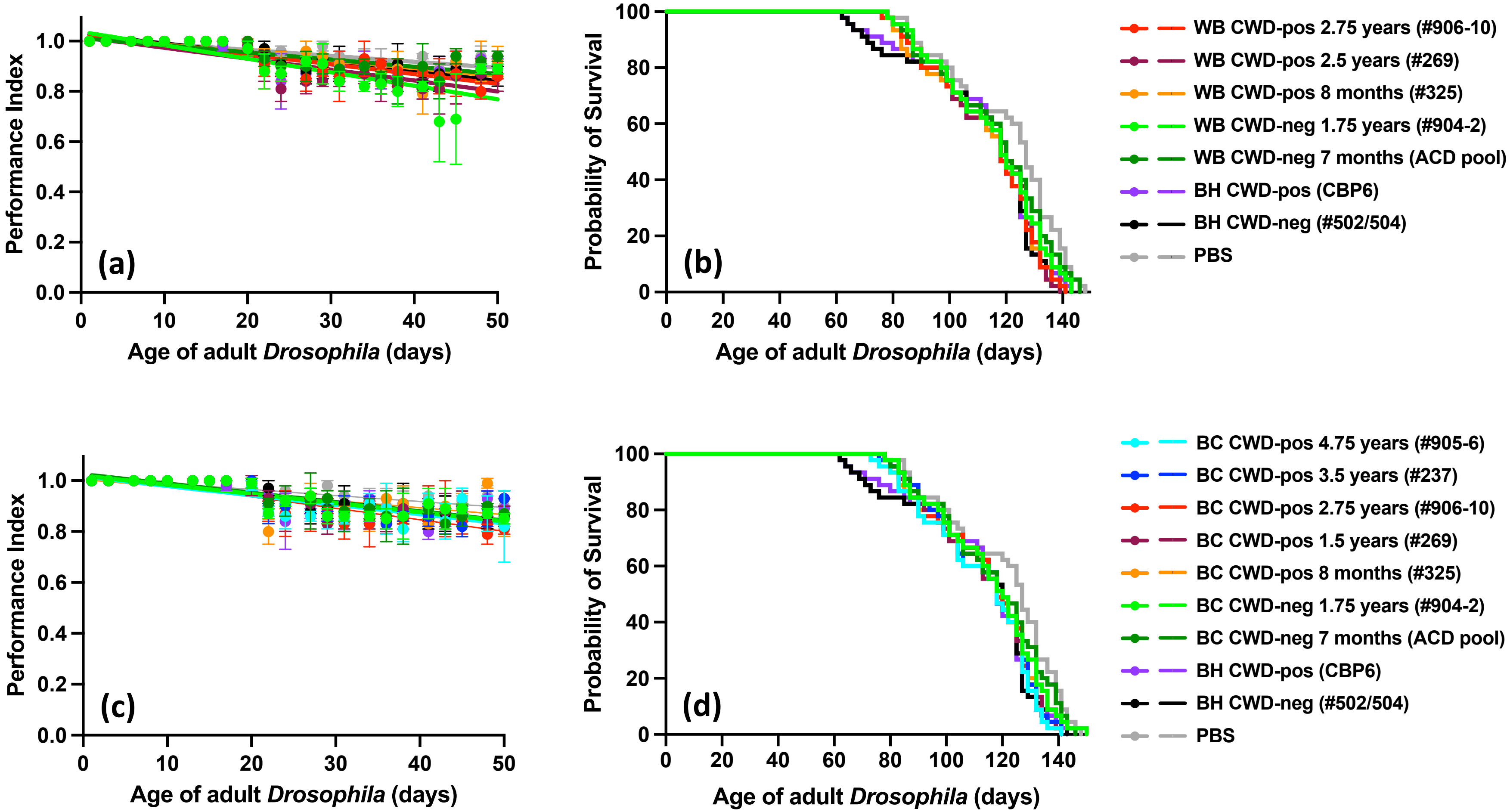

Supplementary Data 11 Legend. *Elav* x 51D *Drosophila* were exposed at the larval stage to a 10^{-2} dilution of defined age samples of whole blood or 10^{6} buffy coat cells from cervids with natural exposure to CWD-pos (CWD-positive) or CWD-neg (CWD-negative) white-tailed deer, or a 10^{-2} dilution of CWD-positive cervid brain homogenate (CBP6), or a 10^{-2} dilution of CWD-negative cervid brain homogenate (cervid #502/504), or PBS. After hatching, adult *Drosophila* were assessed for locomotor activity by a negative geotaxis climbing assay (a) and (c) or for survival (b) and (d). The mean performance index (± SD), expressed as a regression line, is shown for three groups of n=15 flies per time point. For the survival assay the number of surviving flies was recorded three times a week and the data shown as Kaplan-Meier plots. Statistical analysis of Supplementary Data 11 is shown in Supplementary Data 12.
Abbreviations: WB = whole blood; BC = buffy coat; BH = brain homogenate; PBS = phosphate buffered saline.

Supplementary Data 12 Statistical analysis of accelerated decline of locomotor activity and loss of survival in defined age natural CWD-infected whole blood or buffy coat sample-exposed 51D *Drosophila*

Inoculum	Whole Blood		Buffy Coat	
	Locomotor activity (Expt. Supp 11a)	Survival (Expt. Supp 11b)	Locomotor activity (Expt. Supp 11c)	Survival (Expt. Supp 11d)
**8 months old CWD-positive (#325)**	***p*=0.1396**	**118* / *p*<0.2927**	***p*=0.7837**	**118* / *p*=0.3525**
**1.5 years old CWD-positive (#269)**	**N/A**	**N/A**	***p*=0.8741**	**118* / *p*=0.3285**
**2.5 years old CWD-positive (#269)**	***p*=0.8301**	**118* / *p*=0.3634**	**N/A**	**N/A**
**2.75 years old CWD-positive (#906-10)**	***p*=0.3383**	**118* / *p*=0.3992**	***p*=0.4530**	**118* / *p*=0.4136**
**3.5 years old CWD-positive (#237)**	**N/A**	**N/A**	***p*=0.8570**	**118* / *p*=0.3817**
**4.75 years old CWD-positive (#905-6)**	**N/A**	**N/A**	***p*=0.7554**	**118* / *p*=0.2333**
**CWD-positive brain (CBP6)**	***p*=0.7295**	**118* / *p*=0.7780**	***p*=0.7295**	**118* / *p*=0.7780**

Supplementary Data 12 Legend. *Elav* x 51D *Drosophila* were exposed at the larval stage to a 10^{-2} dilution of defined age samples of whole blood or 10^{6} buffy coat cells from cervids with natural exposure to CWD (CWD-positive) or CWD-negative white-tailed deer, or a 10^{-2} dilution of CWD-positive cervid brain homogenate (CBP6), or a 10^{-2} dilution of CWD-negative cervid brain homogenate (cervid #502/504), or phosphate buffered saline (PBS). After hatching, adult *Drosophila* were assessed for locomotor activity by a negative geotaxis climbing assay (see Supplementary Data 11a and 11c) or for survival (see Supplementary Data 11b and 11d). Statistical analysis of the negative geotaxis climbing assay data was performed using an unpaired (two-tailed) Student t test. The Log-rank (Mantel-Cox) test was used to assess the survival assay data. In both analyses, each CWD-positive whole blood or buffy coat treatment group was compared to the 1.75 years old CWD-negative whole blood or buffy coat treatment group from cervid #904-2. The CBP6 CWD-positive brain homogenate treatment group was compared to the CWD-negative brain homogenate treatment group (cervid #502/504). Comparison of whole blood and buffy coat CWD-negative treatment groups (ACD pool and cervid #904-2, respectively), and these groups individually with PBS, were not statistically significantly different ($p>0.05$, data not shown) except 1.75 years old CWD-negative whole blood (cervid #904-2) compared to PBS (Expt. Supp. 11a) ($p=0.0326$) and CWD-negative brain treatment group (cervid #502/504) compared to PBS (Expt. Supp. 11b) ($p=0.0197$). # = cervid identification number; * = median survival time in days of prion treatment group; N/A = not applicable.

Supplementary Data 13 Statistical analysis of accelerated decline of locomotor activity following secondary passage of CWD-infected primary passage cervid PrP *Drosophila*

	2˚ passage - cervid PrP *Drosophila*	
**Inoculum**	**1˚ passage - cervid PrP *Drosophila***	**1˚ passage - 51D *Drosophila***
**Whole blood CWD-positive (#1316)**	***p*=0.0047**	***p*=0.7183**
**CWD-positive brain homogenate (CBP6)**	***p*=0.0002**	***p*=0.8805**
**Assay Control**	**1˚ passage – cervid PrP *Drosophila***	**1˚ passage - 51D *Drosophila***
**CWD-positive brain homogenate (CBP6)**	***p*=0.0065**	***p*=0.6282**

Supplementary Data 13 Legend. Cervid whole blood and brain homogenate was passaged in *Elav* x cervid PrP *Drosophila* or *Elav* x 51D *Drosophila* (primary passage). The inocula at primary passage were the following: whole blood from a white-tailed deer with clinical experimental CWD (CWD-positive blood from animal #1316); whole blood from a CWD-negative white-tailed deer (CWD-negative blood from animal #1437); cervid CWD-positive brain homogenate (CBP6) (CWD-positive brain); brain homogenate from a CWD-negative cervid (CWD-negative brain); or phosphate buffered saline (PBS). Head homogenate was prepared from 40-day old primary passage *Drosophila* and subsequently used to inoculate a fresh batch of *Elav* x cervid PrP *Drosophila* (secondary passage). After hatching, a negative geotaxis climbing assay was performed on secondary passage adult *Elav* x cervid PrP *Drosophila* derived from primary passage in *Elav* x cervid PrP *Drosophila* (see Figure 9a) or from *Elav* x 51D *Drosophila* (see Figure 9b). Statistical analysis of the negative geotaxis climbing assay data was performed using an unpaired (two-tailed) Student t test with CWD-positive treatment groups compared to their respective CWD-negative treatment group. For the assay controls, the CWD-positive brain homogenate treatment group (CBP6) was compared to the CWD-negative brain homogenate treatment group (cervid #502/504). Statistical analysis of median survival times was carried out using Kaplan-Meier plots. Comparisons that are statistically significantly different ($p<0.05$) are shown in red. Comparison of CWD-negative treatment groups with PBS were not statistically significantly different ($p>0.05$, data not shown). # = cervid identification number. 1˚ passage = primary passage. 2˚ passage = secondary passage.

Supplementary Data 14 Statistical analysis of prion seeding activity following secondary passage of CWD-infected primary passage cervid PrP *Drosophila*

Inoculum	1˚ passage	2˚ passage - cervid PrP *Drosophila* Day 5	2˚ passage - cervid PrP *Drosophila* Day 40
**Whole blood CWD-positive (#1316) (Figure 10a)**	**Cervid PrP *Drosophila***	***p*>0.9999**	***p*=0.4667**
**CWD-positive brain homogenate (CBP6) (Figure 10a)**		***p*>0.9999**	***p*=0.0002**
**Whole blood CWD-positive (#1316) (Figure 10b)**	**51D *Drosophila***	***p*>0.9999**	***p*>0.9999**
**CWD-positive brain homogenate (CBP6) (Figure 10b)**		***p*>0.9999**	***p*>0.9999**
**Transmission control**		**1˚ passage day 5**	**1˚ passage day 40**
**CWD-positive brain homogenate (CBP6) (Figure 10c)**		***p*=0.7128**	***p*=0.0002**
**RT-QuIC assay control**			
**CWD-positive brain homogenate (CBP6) (Figure 10e)**		***p*<0.0001**	

Supplementary Data 14 Legend. Cervid whole blood and brain homogenate was passaged in *Elav* x cervid PrP *Drosophila* or *Elav* x 51D *Drosophila* (primary passage). The inocula at primary passage were the following: whole blood from a white-tailed deer with clinical experimental CWD (CWD-positive blood from animal #1316); whole blood from a CWD-negative white-tailed deer (CWD-negative blood from animal #1437); cervid CWD-positive brain homogenate (CBP6) (CWD-positive brain); brain homogenate from a CWD-negative cervid (CWD-negative brain); or phosphate buffered saline (PBS). Head homogenate was prepared from 40-day old primary passage *Drosophila* and subsequently used to inoculate a fresh batch of *Elav* x cervid PrP *Drosophila* (secondary passage). After hatching, prion seeding activity was assessed by RT-QuIC on secondary passage adult *Elav* x cervid PrP *Drosophila* derived from primary passage in *Elav* x cervid PrP *Drosophila* (see Figure 10a) or from *Elav* x 51D *Drosophila* (see Figure 10b). Head homogenates were prepared from 5-day and 40-day old secondary passage *Drosophila* (see Figure 10a and 10b) or primary passage *Drosophila* (see Figure 10c and 10d). Statistical analysis of the RT-QuIC data was performed using an unpaired, non-parametric, two-tailed Mann-Whitney test with CWD-positive treatment groups compared to their respective CWD-negative treatment group. For the transmission and RT-QuIC assay controls, the CWD-positive brain homogenate treatment group (CBP6) was compared to the CWD-negative brain homogenate treatment group (cervid #502/504). Comparisons that are statistically significantly different (*p*<0.05) are shown in red. Comparison of CWD-negative treatment groups with PBS were not statistically significantly different (*p*>0.05, data not shown). # = cervid identification number.
