# Supplementary material for: Successful bioassay of blood from chronic wasting disease-affected cervids in cervid PrP transgenic Drosophila
Source: Acta Neuropathol Commun. 2026 Jul 15;14:211. doi: 10.1186/s40478-026-02372-1 (PMC13651417; doi:10.1186/s40478-026-02372-1)

Bujdoso Supplementary file uncropped original version of Figure 1

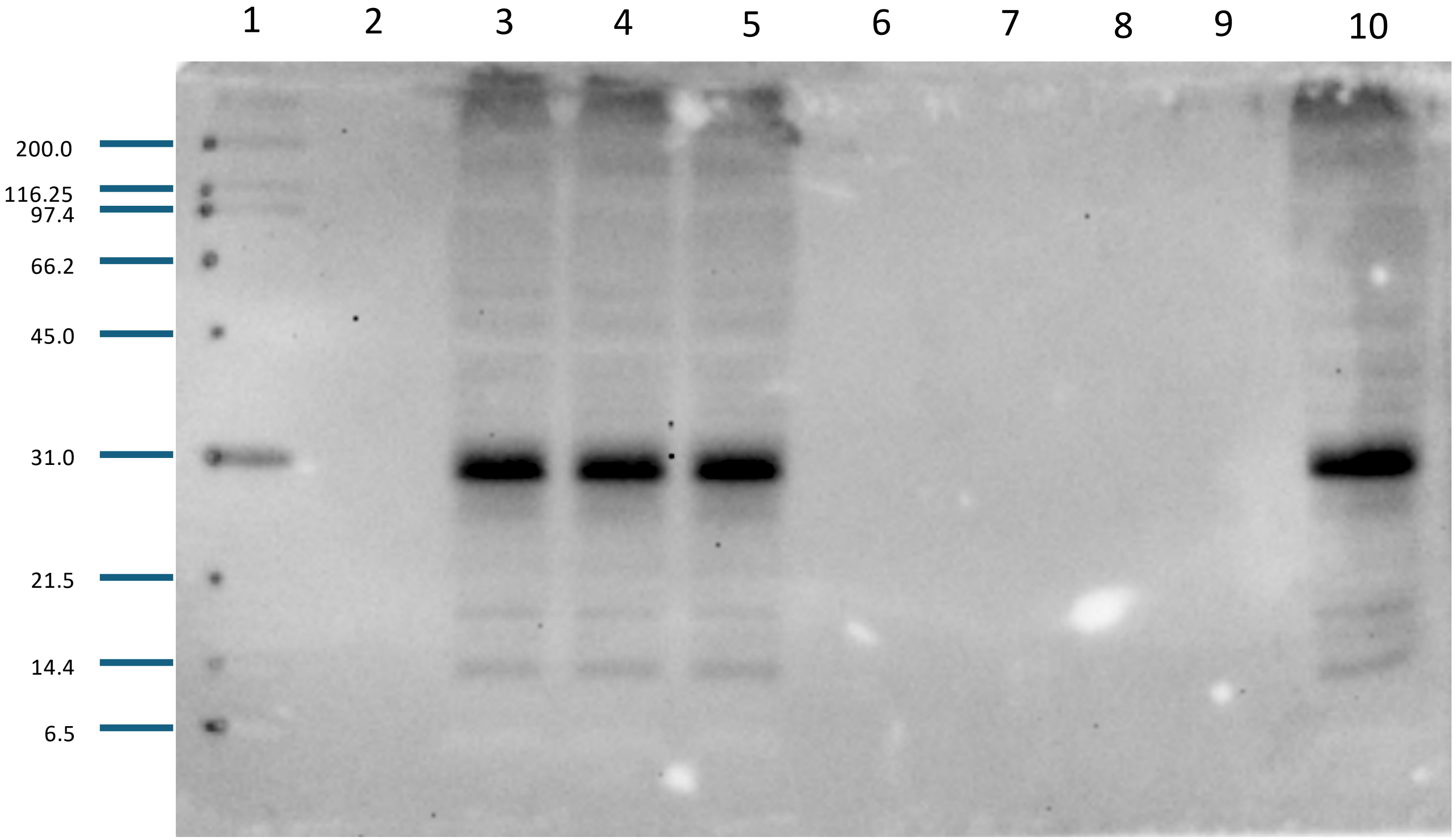

Bujdoso Supplementary file uncropped original version of Figure 1 **brightness 24% and contrast 40%**

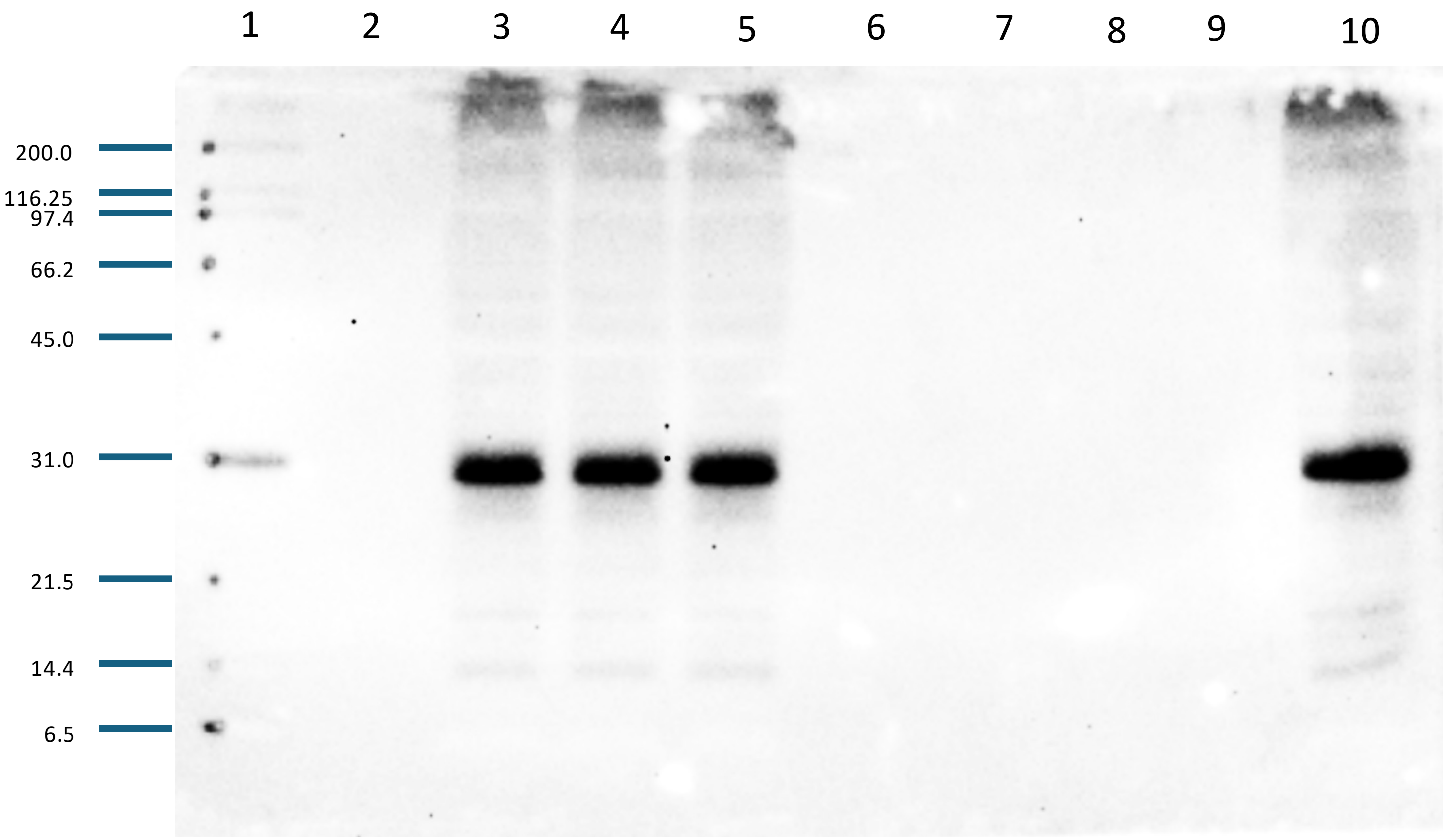

Supplement: Supplementary file 2 [file 40478_2026_2372_MOESM2_ESM.pdf]
